# A Multipronged Bioengineering, Spectroscopic and Theoretical Approach in Unravelling the Excited-State Dynamics of the Archetype Mycosporine Amino Acid

**DOI:** 10.1021/acs.jpclett.4c01254

**Published:** 2024-07-12

**Authors:** Michael Hymas, Sopida Wongwas, Simin Roshan, Abigail L. Whittock, Christophe Corre, Reza Omidyan, Vasilios G. Stavros

**Affiliations:** † Department of Chemistry, University of Warwick, Coventry CV4 7AL, United Kingdom; ‡ School of Chemistry, University of Birmingham, Edgbaston B15 2TT, United Kingdom; § School of Life Sciences, University of Warwick, Coventry CV4 7AL, United Kingdom; ∥ Department of Chemistry, University of Isfahan, 81746-73441 Isfahan, Iran; ⊥ Analytical Science Centre for Doctoral Training, Senate House, University of Warwick, Coventry CV4 7AL, United Kingdom

## Abstract

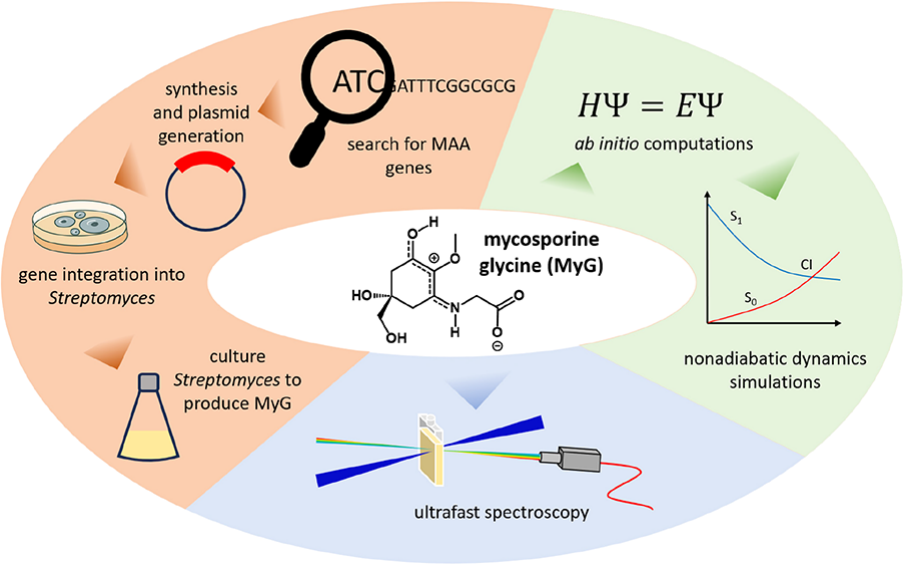

Mycosporine glycine (MyG) was produced by the fermentation of a purposely engineered bacterial strain and isolated from this sustainable source. The ultrafast spectroscopy of MyG was then investigated in its native, zwitterionic form (MyG_zwitter_), via femtosecond transient electronic absorption spectroscopy. Complementary nonadiabatic (NAD) simulations suggest that, upon photoexcitation to the lowest excited singlet state (S_1_), MyG_zwitter_ undergoes efficient nonradiative decay to repopulate the electronic ground state (S_0_). We propose an initial ultrafast ring-twisting mechanism toward an S_1_/S_0_ conical intersection, followed by internal conversion to S_0_ and subsequent vibrational cooling. This study illuminates the workings of the archetype mycosporine, providing photoprotection, in the UV–B range, to organisms such as corals, macroalgae, and cyanobacteria. This study also contributes to our growing understanding of the photoprotection mechanisms of life.

Chemical species capable of providing photoprotection to organisms vary widely in Nature. Such natural products are often produced in complex mixtures and are challenging to isolate in high yields for industrial applications. However, advances in engineering biology open the way to developing sustainable production platforms for high-value chemicals. For instance, DNA information required for the assembly of UV filters such as mycosporine-like amino acids (MAAs) can be introduced in host microorganisms, which then acquire the ability to produce such compounds.^1^

In this article, we focus on mycosporine glycine (MyG) (Figure 1a), a UV filter found in corals, various cyanobacteria, and other species.^2^ MyG is the archetype mycosporine, a class of molecules derived from a cyclohexenone core. The excited state photochemistry of neutral MyG (a model, non-native system) have recently been investigated computationally.^3^ It was revealed that, following excitation to the optically bright S_2_ (^1^ππ*) state, excited population (i) proceeds along a ring-twisting coordinate, rapidly traversing the S_2_/S_1_ conical intersection (CI), (ii) is driven toward a second S_1_/S_0_ CI, along a similar ring-puckering coordinate, where deactivation to the ground state is facilitated by internal conversion (IC), and, finally, (iii) vibrationally cools to recover the initial system.

**Figure 1:**
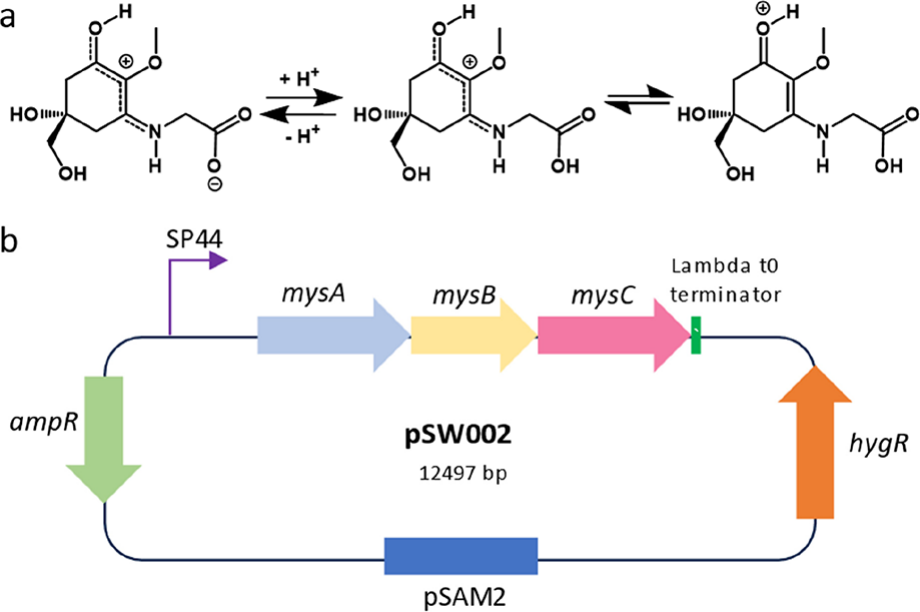
(a) Structures of zwitterionic (left) and protonated MyG (resonance forms, center and right). (b) pSW002 plasmid containing *mysABC* genes under the control of SP44 promoter. Lambda t0 terminator was included to increase transcription rate. [Legend: hygR = hygromycin resistant gene, ampR = ampicillin-resistant gene. pSAM2 is an integrative element that helps integrate the plasmid onto the *Streptomyces* chromosome.]

In this study, a novel bioengineered bacterial producer of MyG has been developed. *Streptomyces albidoflavus* J1074 has been exploited as a bacterial host, and the ultrafast photochemistry of the resulting MyG has been investigated for the first time in its native zwitterionic form, MyG_zwitter_ (Figure 1a). To explicate the ultrafast photochemistry of MyG_zwitter_, we investigate the nonadiabatic dynamics of the computationally tractable MyGH^+^ (Figure 1a), which demonstrates near-identical dynamics to the native MyG_zwitter_. Computation reveals a predominantly barrierless ring-puckering mechanism that drives excited population to the ground state via IC, supporting the experimentally observed photochemistry of MyG_zwitter_, and adds to the library of knowledge surrounding the photoprotection mechanisms of life.

Initially, *Streptomyces albidoflavus* J1074 was chosen as a bacterial host to clone and express the *mysA*, *mysB*, and *mysC* genes known to direct MyG biosynthesis (further detail on *Streptomyces* and the synthesis procedure can be found in the Supporting Information (SI), Section A-1/2). *Streptomyces* bacteria, known for their ability to produce a variety of specialized metabolites, are commonly used as hosts for heterologous expression.^4^ The amino acid sequences of the *Rhodococcus fascians* D188 MysA, MysB, and MysC enzymes, known to direct the biosynthesis of MyG, were used to design synthetic DNA sequences optimized for expression in *S. albidoflavus*. The synthetic *mysABC* gene cluster was cloned under the control of a strong constitutive promoter (SP44) in a *Streptomyces* integrative plasmid (pJCC025), generating plasmid pSW002 (Figure 1b).

Strains containing the expected genes were selected to culture further and inoculated in media at 30 °C for 5 days. The supernatant was subsequently centrifuged and filtered for ultraviolet–visible (UV-vis) absorption analysis; molecules were identified with absorption maxima at ∼310 nm, which is expected for MyG at biological pH.^5^

To confirm the identity of the produced molecules, filtrate of the supernatant was analyzed with liquid chromatography–mass spectrometry (LC-MS) methods, known to be highly sensitive for mycosporine analysis, yielding a component whose *m*/*z* value (246.972, protonated) agreed with that predicted for MyG (Figure 2).^6^ From knowledge of its extinction coefficient (28 100 M^–1^ cm^–1^), it was determined that ∼150 mg/L MyG was produced after 5 days incubation in media (see Sections A-5/6/7 in the SI); it is possible that, after longer durations, this value could be increased.^5,7,8^ MyG was subsequently purified using reversed-phase chromatography separation from the culture supernatant in high purity for use in spectroscopy experiments.

**Figure 2:**
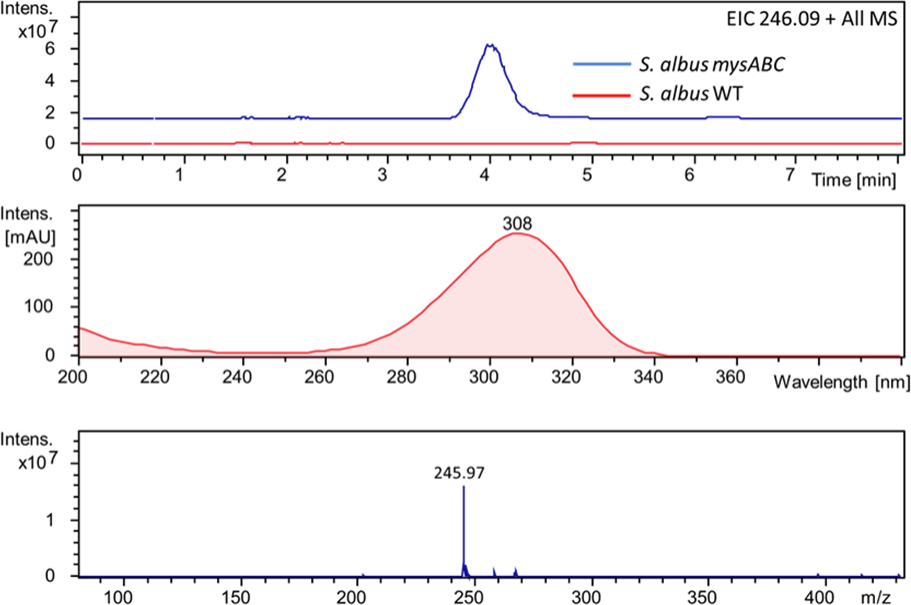
LC-MS analysis of culture supernatants of *S. albidoflavus* engineered to express *mysABC* genes. (Top) Extracted ion chromatogram at 246.09 (±0.5) for the *mysABC* (blue trace) and wild-type (red trace) strains. (Middle) UV spectrum of MyG detected in the engineered strain with λ_max_ at 308 nm (∼310 nm) at a retention time of 4 min. (Bottom) Mass/charge (*m*/*z*) of MyG detected in positive-ion mode at 245.97.

UV-vis absorption spectra were taken of extracted MyG in a solution buffered at pH 5.0 and in a solution of 0.1 M HCl (pH 1.0) (further details on spectroscopy experiments can be found in Section B in the SI); these are shown in Figure 3. The UV-vis spectra at these two pH values were similar, with a shift of only ∼3 nm between maxima of MyGH^+^ (302 nm) and MyG_zwitter_ (305 nm). Given this modest spectral shift, we anticipate that the photochemistry of MyGH^+^ and MyG_zwitter_, following excitation to the same excited state, is similar at pH 1.0 and 5.0, which we verified with femtosecond transient electronic absorption spectroscopy (fs-TEAS, see below).

**Figure 3:**
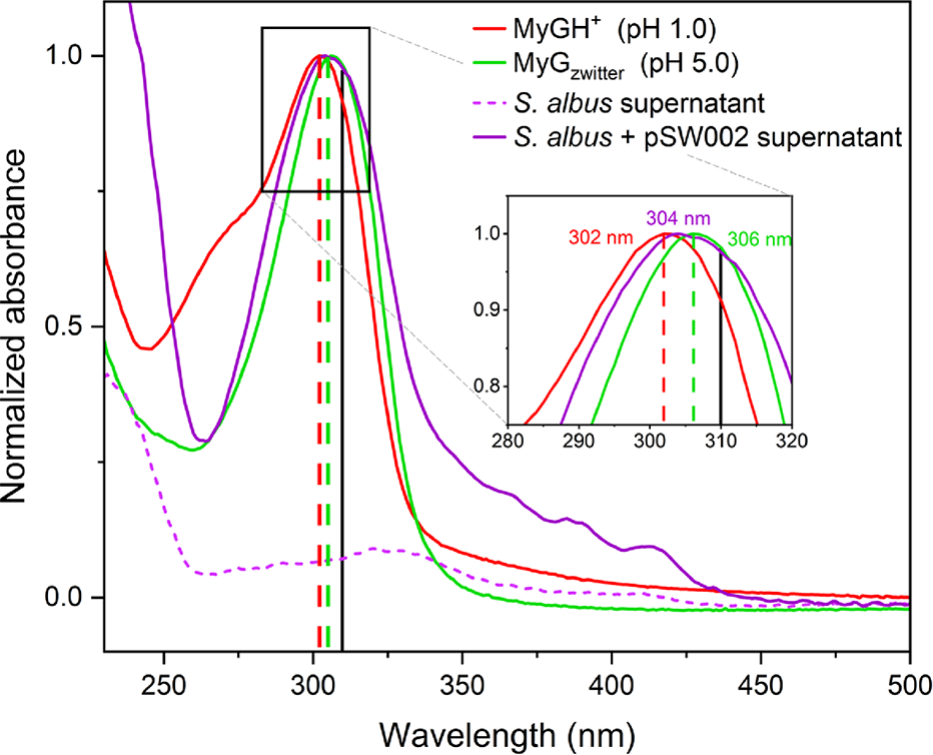
Normalized UV-vis spectra of the supernatant of *S. albidoflavus* WT (dotted purple), *S. albidoflavus* with *mysABC* cluster (solid purple), and purified MyG at pH 1.0 (red) and 5.0 (green); peak absorption wavelengths (λ_max_) for each pH (dashed red and green) and the “pump” wavelength selected for use in our transient absorption spectroscopy experiments (solid black) are also shown. Inset shows a magnified range of 280–320 nm, highlighting the difference in absorption maxima for three MyG-containing solutions (purple, red, and green numbers).

In interpreting our steady-state spectroscopy results, we devised a computational approach beginning with a comprehensive search for the lowest energy conformers of MyGH^+^ and MyG_zwitter_.^3^ Our studies revealed local minima on the ground-state potential energy surface (PES) of both species; Figure 4 includes the optimized structures of MyGH^+^ and MyG_zwitter_. Details on all computations can be found in Section C in the SI. Our calculations revealed that protonation at the cyclohexenone oxygen (labeled in Figure 4a, hereafter referred to as “O_9_”) generates the most stable MyGH^+^ system. Further computations were then performed on this isomer of MyGH^+^, including ground- and excited-state geometry optimization (Figure 4) and determination of photophysical properties.

**Figure 4:**
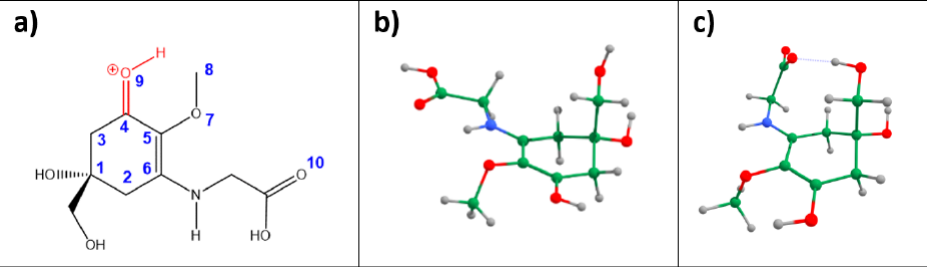
(a) Structure and atomic numbering scheme of the most stable protonated isomer of MyG. (b) optimized geometry of MyGH^+^, (c) optimized geometry of MyG_zwitter_, determined at the RI-MP2 theoretical level. For the determination of MyG_zwitter_, an implicit water solvent model was used.

Computationally, because of the highly flexible structures, identification of the lowest energy conformers of MyGH^+^ and MyG_zwitter_ was the first challenge in this work. To tackle this, a metadynamics (MTD) conformational search was employed to determine the lowest-lying conformers and finally the most stable structure of both systems (see Section C-2 in the SI).^9^ The UV absorption spectra were then simulated and compared to the experiment. Our theoretical results generated comparatively similar geometries for MyGH^+^ and MyG_zwitter_. The cyclohexenone ring in both systems is protonated at O_9_, and the only slight structural differences between these two systems are related to the carboxylic side chain: it is protonated in MyGH^+^ and is a carboxylate in MyG_zwitter_ (see Figure 4). Semblance in electronic structure between these two systems (Figure S13) demonstrates that protonation does not significantly affect the electronic states (and thereby UV-vis absorption).^5^ We therefore suggest that the photochemistry of MyG_zwitter_ and MyGH^+^ should be broadly similar, enabling us to focus our attention on the computationally tractable MyGH^+^ for the nonadiabatic (NAD) simulations discussed later.

In Table 1, we present calculated vertical transition energies and respective oscillator strengths for the four lowest-lying singlet excited states (S_1_–S_4_) of MyGH^+^ (see the SI for a similar treatment of MyG_zwitter_). We employed different levels of theory: time-dependent density functional theory (TD-DFT), using the ωB97XD functional, RI-ADC(2), and MS-CASPT2 (where MyGH^+^ is microsolvated with three water molecules, MyGH^+^·3H_2_O). Explicit water did not affect the excitation energies for MyGH^+^ calculated with TD-DFT and RI-ADC(2), so these are reported using an implicit solvent model (see the SI for details). The ωB97XD functional was employed due to its success in describing the electronic energies and nonradiative deactivation processes in similar systems.^3,10−15^ Photoexcitation at λ_max_ of MyGH^+^, determined as 310 nm (4.00 eV), corresponds to population of the bright S_1_ state (1^1^ππ*), which agrees with our experimental results. The consistency between selected theoretical levels (ab initio and TD-DFT), indicates reliability in describing the photochemistry of the MyGH^+^ system. The similarity between UV absorption of MyG at pH 5.0 and 1.0 (both ∼310 nm) agrees with previous findings, and the closeness to the calculated vertical excitation energies identifies these two protonation states of MyG (MyG_zwitter_ and MyGH^+^) as the predominant absorbing species in aqueous solutions.^5^ The conjugated system is consistently protonated across the pH range, leading to an efficient charge resonance.

**Table 1:** Vertical Transition Energies and Oscillator Strength for MyGH^+^ at Different Levels of Theory^a^

	Transition Energy/eV					
	ADC (2)		TD-ωB97XD		MS-CASPT2	
excited state	gas phase	cosmo/water	gas phase	PCM/water	gas phase	MyGH^+^·3H_2_O
S_1_ (ππ*)	3.99 (0.3453)	4.00	4.32 (0.2890)	4.36	4.05 (0.4580)	4.03
S_2_ (*n*π*)	5.03 (0.0785)	5.20	5.33 (0.0161)	5.56	5.40 (0.1898)	5.05
S_3_ (*n*π*)	5.21 (0.0261)	5.37	5.44 (0.0985)	5.68	5.54 (0.0840)	5.72
S_4_ (*n*π*)	5.96 (0.0015)	6.16	6.02 (0.0191)	6.15	7.04 (0.1608)	6.99

a The values in parentheses represent oscillator strength.

Higher energy electronic transitions in Table 1 (i.e., S_2_–S_4_) have been assigned as ^1^*n*π* states, which contributes little to the UV-vis absorption spectrum of MyGH^+^, due to their low oscillator strengths. Note that the inclusion of an implicit solvent model does not significantly affect the S_1_ transition energy, although it slightly destabilizes the lowest ^1^*n*π* states. Molecular orbitals contributing to the four lowest lying electronic transitions (S_1_–S_4_) can be found in Section C in the SI.

As discussed previously, we hypothesize that the similarity in the spectral features between MyGH^+^ and MyG_zwitter_ suggests that the dynamics following photoexcitation to the S_1_ excited state in each is also similar. We now test this hypothesis. fs-TEAS studies were undertaken on MyG at pH 5.0 (Figure 5) and 1.0 (Figure S2) to compare excited-state ultrafast processes under these conditions.

**Figure 5:**
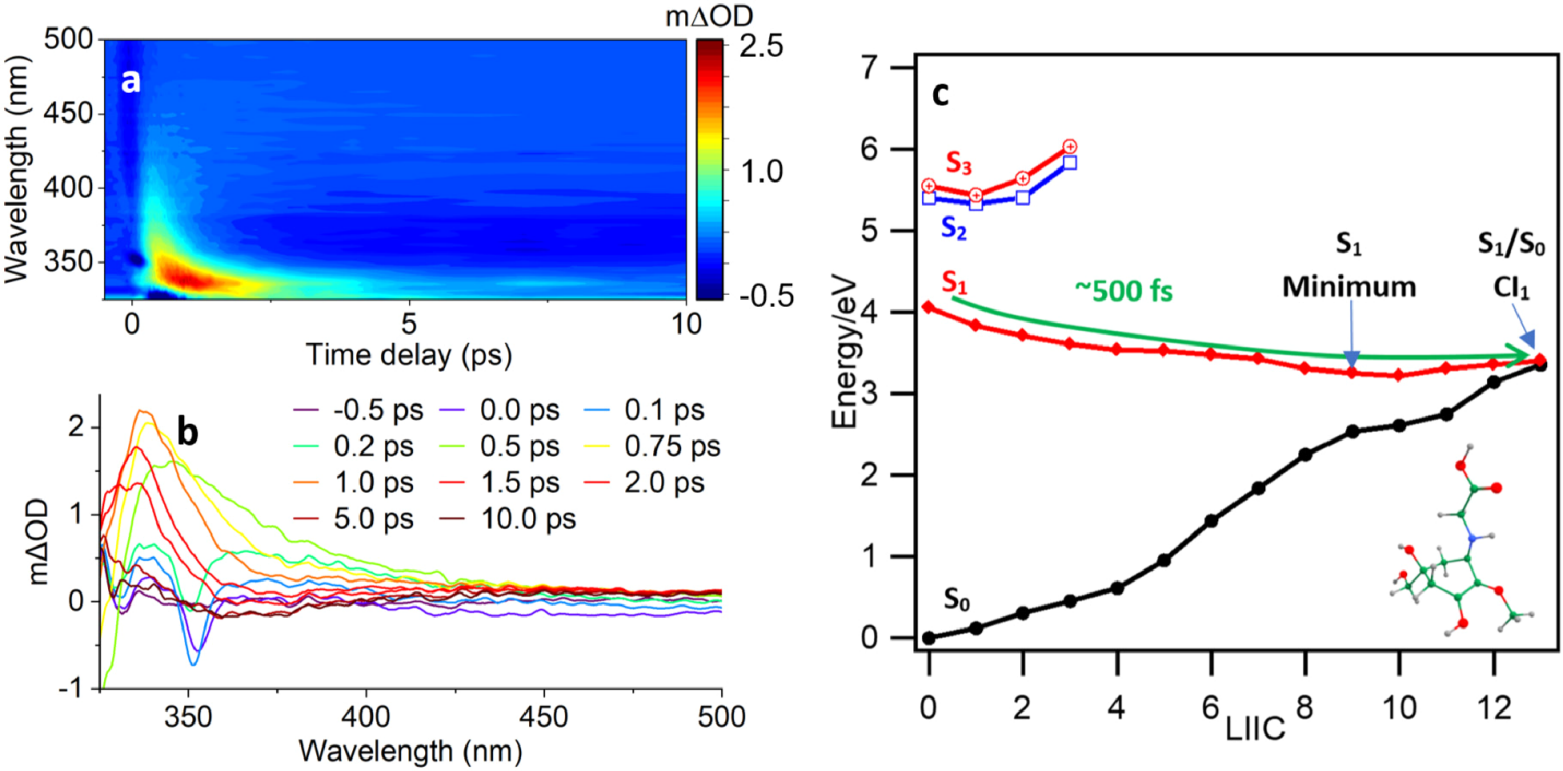
(a) Collected TAS and (b) selected time slices for MyGH^+^ at pH 5.0 following photoexcitation at 310 nm. (c) PE profiles of the ground (black) and S_1_ excited (red) states of MyGH^+^ calculated at the MS-CASPT2/SA-CASSCF(6,6)/cc-pVDZ level of theory along the LIIC reaction path; the inset represents the optimized geometry of the S_1_/S_0_ CI_1_.

Lifetimes extracted from global analysis of collected transient absorption spectra (TAS) obtained after photoexcitation at 310 nm in both cases are summarized in Table 2.^16^ From previous studies on commensurate systems (along with our NAD simulations, below), we assign lifetime τ_1_ to rapid movement of the excited-state wavepacket out of the Franck–Condon (FC) region, followed by propagation along the S_1_ PES toward a S_1_/S_0_ CI.^17^ This is evidenced by strong excited state absorption (ESA) at ∼340 nm, which blue-shifts with an increasing time delay as the excited state relaxes. Stimulated emission (SE) is also seen briefly near time-zero, which has been described in similar systems as occurring from the FC region on the S_1_ PES.^18^ τ_2_ and τ_3_ then respectively correspond to vibrational cooling in the ground state (S_0_), and a photoproduct signal extending beyond the bounds of our experimental window (10.0 ps). The vibrational cooling observed in the ground state is extremely fast, attributed to strong interactions afforded by the ionic character of the native zwitterion (and protonated species) and the solvent. It is evident from Figure 5a that a negative SE signal persists beyond 10 ps, suggesting that some population remains in an excited state after the primary deactivation coordinate is completed. This may explain the observed degradation of MyG following sustained irradiation (see Figure S7).

**Table 2:** Lifetimes Extracted from Global Analysis of Collected TAS for MyG at pH 5.0 and 1.0, Following Photoexcitation at 310 nm

system	τ_1_ (fs)	τ_2_ (ps)	τ_3_ (ps)
pH 5.0 (MyG_zwitter_)	450 ± 100	1.07 ± 0.10	>10.0
pH 1.0 (MyGH^+^)	510 ± 80	1.40 ± 0.08	>10.0

We deduce from fs-TEAS that the photochemistry of MyGH^+^ and MyG_zwitter_ are equivalent. To rationalize ultrafast processes identified from experiment, we have performed further calculations. Accurate calculations for MyG_zwitter_ require the use of an explicit solvent model, which are computationally expensive. As such, we selected the more tractable MyGH^+^, implementing the affordable implicit solvent model. In searching for possible S_1_/S_0_ crossings, two optimized CIs were located using the SA-CASSCF (6,6)/cc-pVDZ theoretical model (see Section C in the SI for structures and further discussion). Both CIs are reached via ring-twisting coordinates; out-of-plane deformation of the six-membered ring from C_6_–C_3_ results in CI_1_, while CI_2_ arises from ring-twisting in the C_2_–C_4_ region (Figure S12). These nuclear motions facilitate an aborted isomerization about the intracyclic C_5_=C_6_ double bond, which is frustrated due to structural constraints effected by the cyclohexenone moiety (see Section C in the SI for details) and are reminiscent of those predicted in the deactivation of photoexcited ππ* states of cytosine and uracil nucleobases.^19,20^

The excited-state topography of MyGH^+^, connecting the Franck–Condon region to the optimized CI_1_ along the seam determined by linear interpolation of internal coordinates (LIIC) is shown in Figure 5c. As shown, the S_1_ PES exhibits a decreasing and barrierless feature, approaches a local S_1_ minimum, and then continues along the reaction coordinate where S_1_/S_0_ curve crossing (CI_1_) is predicted. In addition, the slight out-of-plane distortion of the cyclohexenone ring mildly stabilizes the S_1_ excited state. This curve crossing provides an essential route for ultrafast deactivation to the ground state via IC. Since the predicted CI_1_ is located significantly lower than the S_1_ vertical transition energy (3.0 eV versus 4.05 eV at the MS-CASPT2 level), one can envisage the S_1_ population approaching the CI without hindrance. The role of CI_2_ in the photodynamics of MyGH^+^ is discussed in Section C in the SI.

To model the time evolution of MyGH^+^ along the above-mentioned pathways, and therefore confirm assignments from fs-TEAS, we performed excited-state NAD simulations using TD-DFT (ωB97XD/cc-pVDZ level, see Section C in the SI). This cost-effective TD-DFT model was selected for its ability to determine electronic transition energies for MyGH^+^ (Table 1) and elucidate photodynamics for similar systems.^3,11,12,15,21,22^ The time taken to reach the crossing point, calculated as ∼500 fs averaged over all trajectories, was assumed to correspond to the lifetime for IC.^12,15,23−25^ All trajectories were analyzed, taking dynamical geometry alterations undergone into account. It was found that 74% of relaxation trajectories were based on ring puckering in the C_6_–C_3_ region, while the remaining relaxed by ring deformation in the C_2_–C_4_ region. Therefore, ∼75% of excited MyGH^+^ relax by accessing CI_1_ and ∼25% by CI_2_. As in Figure S14, the barrier in the S_1_ PES before CI_2_ potentially explains why this crossing point is less favored in population deactivation.

Population analysis from the NAD simulations is consistent with our ab initio interpretations provided by PES topographies and, importantly, is in remarkable agreement with the experimentally determined lifetime for the IC, given by τ_1_ in Table 2 (cf. 500 fs NAD simulations vs ∼510 fs for MyGH^+^). The proposed relaxation mechanisms, shown by Figure 5c for CI_1_, provide efficient deactivation coordinates, driving the excited-state population to the ground via ultrafast IC.

In summary, by combining bioengineering, femtosecond transient electronic absorption spectroscopy, and nonadiabatic simulations, we have elucidated, for the first time, the photodynamics of the archetype mycosporine, MyG, in its native zwitterionic form. Photoexcitation of MyG_zwitter_ and MyGH^+^ to the S_1_ state leads to fast relaxation via internal conversion, mediated by ring puckering, to an S_1_/S_0_ conical intersection. Subsequent (highly efficient) vibrational cooling in the ground state occurs. The results presented are a vital addition to existing knowledge of the photoprotection mechanisms of life. Given the importance of structure–dynamics–function relationships in a multitude of fields, our work on the archetype MyG provides valuable insight into the application of similar mycosporines as effective molecular heaters, which, broadly speaking, can be exploited for many photothermal applications, including biomimetic UV radiation filters for sunscreen application.

## Supporting Information Available

Details of the genetic synthesis process and characterization of MyG from *S. albidoflavus* (Section A), details of the transient absorption experiments, including solvent responses, evolution associated difference spectra and residuals from global fitting, photostability results (Section B), and computational details, including a description of methods for obtaining optimized ground state geometry, electronic transition energies, conical intersections, potential energy surfaces and nonadiabatic dynamics (Section C) (PDF)
