# Supplementary material for: A Multipronged Bioengineering, Spectroscopic and Theoretical Approach in Unravelling the Excited-State Dynamics of the Archetype Mycosporine Amino Acid
Source: J Phys Chem Lett. 2024 Jul 12;15(29):7424–9. doi: 10.1021/acs.jpclett.4c01254 (PMC11284840; doi:10.1021/acs.jpclett.4c01254)
Supplement: Supplementary file 1 — jz4c01254_si_001.pdf [file jz4c01254_si_001.pdf]

Supplementary Information - A Multipronged Bioengineering, Spectroscopic and Theoretical Approach in Unravelling the Excited State Dynamics of the Archetype Mycosporine Amino Acid

Michael Hymas† [1, 2], Sopida Wongwas† [3], Simin Roshan† [4], Abigail L. Whittock[1,5], Christophe Corre[1,3], Reza Omidyan[4], Vasilios G. Stavros[2]

[1]*Department of Chemistry, University of Warwick, Coventry CV4 7AL, United Kingdom*

[2]*School of Chemistry, University of Birmingham, Edgbaston, B15 2TT, United Kingdom*

[3]*School of Life Sciences, University of Warwick, Coventry CV4 7AL, United Kingdom*

[4]*Department of Chemistry, University of Isfahan, 81746-73441, Isfahan, Iran*

[5]*Analytical Science Centre for Doctoral Training, Senate House, University of Warwick, Coventry, CV4 7AL, United Kingdom*

Section A

A-1) General Information

Streptomyces is a genus of Gram-positive bacteria that are known for their ability to produce a wide variety of specialised metabolites, including antibiotics.[1] Given the important role *Streptomyces* play in the pharmaceutical industry, there has been much interest in using genetic engineering to improve the yield of desired products generated by these bacteria. The most widely used *Streptomyces* strains as heterologous hosts for the expression of biosynthetic gene clusters (BGC's) are derivatives of *S. coelicolor*, *S. lividans*, *S. albidoflavus*, *S. avermitilis* etc.[2]

In this study, *S. albidoflavus J1074* was chosen for use in the experiments. *S. albidoflavus* was initially used as a readily transformable host for cloning and expression of *Streptomyces spp.* genes.[3] The widely-used strain *S. albidoflavus J1074* was later developed by mutating the *S. albidoflavus* G strain to lack the SalI restriction modification system.[4] *S. albidoflavus* has become one of the most successful strains for heterologous expression of BGC's, and although *S. albidoflavus J1074* is frequently used, few efforts have been made to enhance its features.[2,5,6]

A-2) Synthesis process

A RiboJ insulator was used to normalise transcripts, reduce variation in the 5' UTR's and prevent genetic circuit context dependence. The 75-nucleotide sequence of the RiboJ was obtained from Clifton *et al.* and added between the SP44 promoter and the SR41 RBS (Ribosome binding site).[7,8]

Synthetic RBS binding sequences were obtained from Bai *et al.*[9] The SR41 RBS (5'-TCTAAGTAAGGAGTAGGCTG-3') was added between the SP44 promoter and the first open reading frame of the *mysA* gene.

In this research, *mysABC* gene clusters from *Rhodococcus fascians* D188 were employed. The *mysABC* gene cluster was then codon-optimised using Build Optimisation Software Tools (BOOST). Desmethyl-4-deoxygadusol synthase (DDGS), which is expressed by the *mysA* gene, catalyses the cyclization of sedoheptulose 7-phosphate (SH-7P) to desmethyl-4-deoxygadusol, which is then methylated by an O-methyltransferase, coding *mysB* to produce 4-deoxygadusol (4-DG). All MAA gene clusters produce the *mysC* enzyme, which links an amino acid (often glycine) to 4-DG to build the compound oxomycosporine. Except for fungus, some organisms (such as bacteria, algae, and marine invertebrates) also possess the fourth gene, *mysD*, which links a second amino acid to form an MAA.[10]

Addition of the lambda t0 terminator was intended to help increase the recycling rate of ribosome and polymerase, resulting in higher product yield.

A-3) Assembly

mysABC genes were synthesised and then amplified by polymerase chain reaction (PCR) with a primer pair SW018 01 FWD *mysABC* and SW018 02 REV *mysABC* (Table S1). Q5 High-Fidelity 2X Master Mix (NEB) was used. pJCC025 were used as vector plasmids, as shown in Figure S1. Golden Gate Assembly was then performed; the reaction mixture contained: 0.5 µL Esp3I (NEB), 0.5 µL T4 ligase, 0.5 µL T4 ligase buffer, 1 µL of 1 mg/mL BSA + 10% PEG-3350, 50 fmol of *mysABC* DNA part, 25 fmol of pJCC025 plasmid backbone, and made up to 10 µL with water. The amplification of plasmids containing the MAA gene cluster was performed in *E. coli* TOP10. The bacteria were grown overnight at 37 °C and Miniprep was undertaken to extract the DNA contents.

A-4) Characterisation of *S. albidoflavus J1074* pSW002: attB

The pSW002 plasmids from *E. coli* TOP10 were extracted through miniprep using Monarch® Plasmid Miniprep Kit and then transformed into *E. coli* ET12567 (pUZ8002). The resultant plasmids were conjugated into *Streptomyces albidoflavus J1074*.

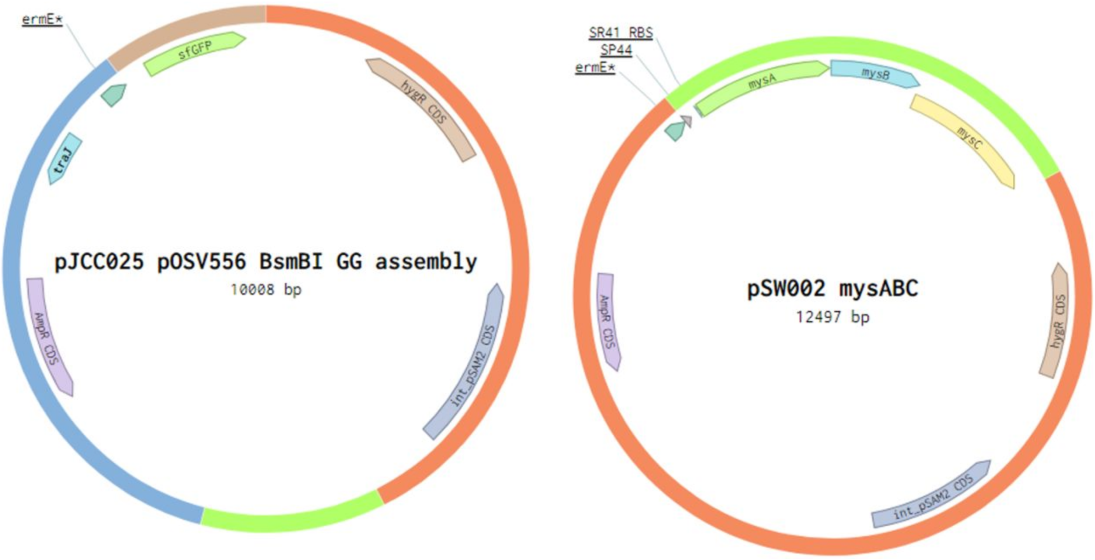

Figure S1. pJCC025 integrative backbone plasmid (left) and pSW002 plasmid with *mysABC* genes (right).

Table S1. Primers used.

Primer	5'-sequence-3'
SW018 01 FWD *mysABC*	CGTGTTCACATTCGAACCGC
SW018 02 REV *mysABC*	GATGATAAAAAACGCCCGGCGGC
SW20_01 FWD bb	TTCGTTCTACCATCGACACCACCACG
SW20_01 REV bb	GTTCCACTGAGCGTCAGAC

Subsequently, gDNA of each strain was extracted to confirm the presence of selected genes in strains. The primers used, SW20_01 FWD bb and SW20_01 REV bb (Table S1), were designed to target the backbone of the vector due to the similarity between *mysABC* genes and *Streptomyces* DNA.

A-5) Mycosporine glycine production

10 µL of *S. albidoflavus J1074* pSW002::attB strain was inoculated in 20 mL R5A media in baffled flasks at 30°C for 5 days. After 5 days, pellets and supernatants were separated by centrifugation at 4000 rpm for 20 minutes. The supernatants were then filtered by 0.2 µm filters, and the filtered supernatants analysed *via* UV-vis measurement using a NanoDrop (Thermo Fisher Scientific). The UV-visible absorbance profile showed absorbing

molecules at 310 nm. To confirm the identity of the compound produced, liquid chromatography–mass spectrometry (LC-MS) methods were used to provide molecular formulae for the absorbing molecule.

A-6) LC-MS analysis of Mycosporine glycine

After filtering the supernatants, the flash chromatography machine Biotage Selekt (Biotage®), was used to select solutions with appreciable absorption in the ~310nm region. 5% methanol in water was used as solvent. This was then analysed *via* LC-MS. LC-MS results showed that the compounds with absorption maxima at 310 nm had the (protonated) m/z value of 246.0972, which corresponds to molecular weight 245 $gmol^{-1}$. In the same dataset, the presence of 4-DG, a precursor molecule to MAAs (discussed *supra*), was detected.

A-7) Concentration of Mycosporine glycine

The concentration of MyG in the supernatant was calculated using the Beer-Lambert law, with the tabulated molar extinction coefficient for MyG (ε = 28100 $M^{-1}cm^{-1}$):

$$c = \frac{A}{\varepsilon L} \quad (1)$$

where A is absorption (approximately 1.788 a.u.), L is path length of attenuator (0.1 cm), the concentration c was determined as 156 mgL^{-1}.[11]

Section B

B-1) Mycosporine glycine solutions

MyG was maintained at pH 5.0 using a sodium acetate/acetic acid buffer. It was not feasible to buffer MyG at pH 1.0, so a 0.1M HCl solution was made (pH 1.0) and the MyG added; pH testing the resultant solution confirmed that the solution was still at pH 1.0-2.0, the pH range identified as that corresponding to protonated MyG.[11]

B-2) Femtosecond transient electronic absorption spectroscopy (fs-TEAS)

Details on the experimental set-up for a general femtosecond transient electronic absorption spectroscopy (fs-TEAS) experiment can be found elsewhere[12], but a summary of our method and modifications is provided herein.

Unlike in previous measurements using a similar fs-TEAS set-up[12], where a solution is typically circulated using a flow-through cell, each sample in this study was prepared in a 1 mm cuvette mounted onto a translating stage. This was due to scarcity of the high-purity MyG sample prepared from reverse-phase chromatography separation, and thereby our inability to make a sufficient volume of solution for circulation.

3 W, 1 kHz femtosecond laser pulses with central wavelength 800 nm were generated by a regenerative amplifier (Spitfire XP, Spectra-Physics) seeded using a Ti:sapphire oscillator (Tsunami, Spectra-Physics). This pulse train was split into three equivalent beams, two of which are utilised for fs-TEAS; one was used to generate 310 nm 'pump' pulses *via* an optical parametric amplifier (TOPAS-C, Spectra-Physics). Another is divided into two beams of 0.95 W and 0.05 W, the latter of which is focussed into a translating CaF_2 window to produce a ~315-740 nm supercontinuum 'probe' pulse. The probe pulse polarisation was kept at magic angle (54.7°) with respect to the pump pulse polarisation, to negate molecular rotational orientation effects on observed dynamics.[13] Pump-probe delay was varied by reflecting the probe pulse off a gold retroreflector mounted on a motorised delay stage. After passing both beams through the sample cuvette, the probe pulse was collimated and focussed into a fibre-coupled spectrometer (AvaSpec-ULS1650F, Avantes) by a CaF_2 lens. Changes in optical density (OD) were then determined from the 'un-pumped' and 'pumped' samples. Fs-TEAS scans at each time delay were repeated a minimum of 10 times for each sample.

Pump-probe time delays between -0.5 and 10 ps were selected for fs-TEAS experiments (as opposed to including the full 1.8 ns afforded by the translation stage-mounted gold retroreflector). This was to minimise the effects of beam irradiation on analyte degradation, again considered for reasons of sample scarcity. It was determined that photochemistry of importance to this study was effectively complete within 10 ps following pump excitation, as evidenced by the steady ΔOD signal observed from ~5 ps to 10 ps in collected TAS.

Collected TAS and selected time slices for MyG at pH 1.0 following photoexcitation at 310 nm is presented in Figure S2.

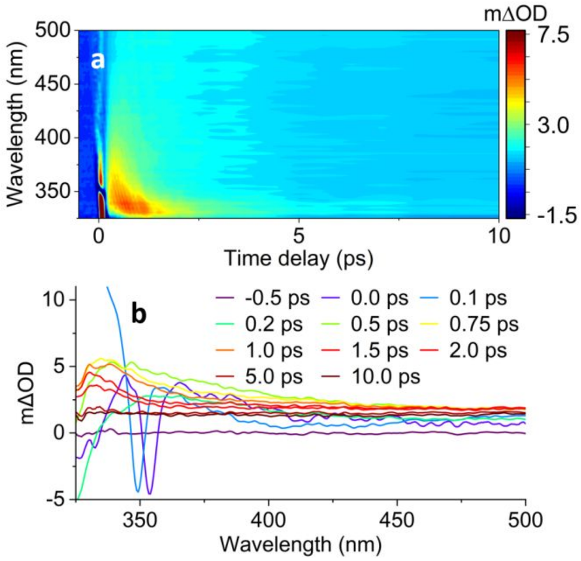

Figure S2. Collected (chirp corrected) TAS (a) and selected time slices (b) for $MyGH^+$ at pH 1.0 following photoexcitation at 310 nm. The probe spectral window has been truncated (as in Figure 5) to focus on spectral features of importance to MyG's excited state dynamics.

B-3) fs-TEAS Solvent response

Equivalent fs-TEAS scans (pump: 310 nm) were taken for pure pH 1.0 and 5.0 solutions without MyG to evaluate an experimental instrument response for detection of ultrafast signals. Traces at wavelengths exhibiting maximum ΔOD signal (see Figure S3) were fitted using a frequency-dependent cross-correlation function F_{cc} from Kovalenko *et al*:[14]

$$F_{cc} \sim \exp\left(\frac{-(t+t_0(\omega))^2}{\tau^2}\right) + offset \qquad (2)$$

where t is the pump-probe time delay, $t_0(\omega)$ is the temporal overlap between the pump and probe pulses, τ is the (approximate) pump pulse duration and *offset* is a correction term.

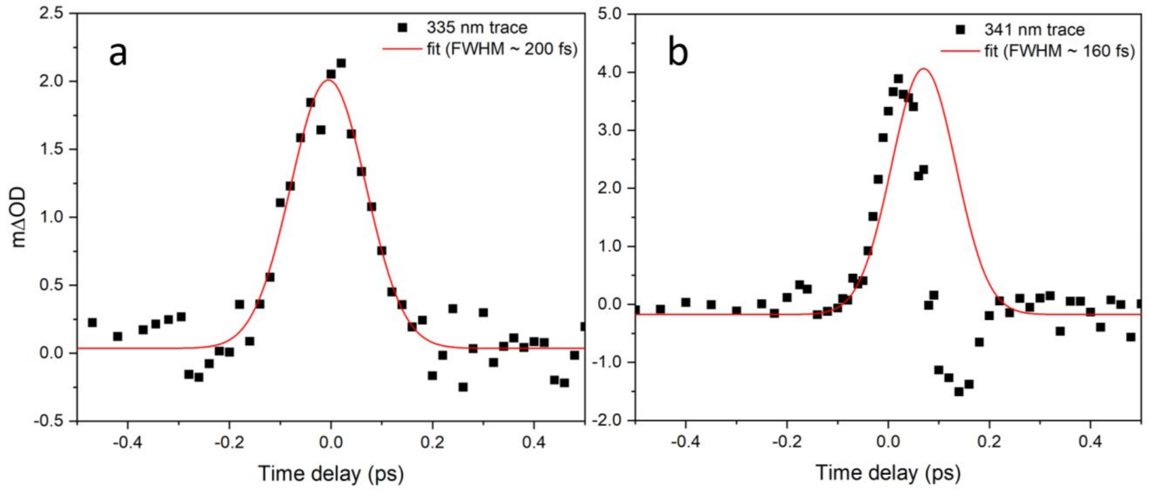

Figure S3. Chirp-corrected TEAS trace of pH 5.0 solution probed at 335 nm (a) and pH 1.0 solution probed at 341 nm (b). The extracted FWHM for F_{cc} is 200 and 160 fs for each system respectively.

For the pH 5.0 solution, the broad F_{cc} FWHM is assigned to time-zero artefacts[15] convoluted with the solution's cross-correlation response.

Where errors output from computing lifetimes *via* our global fitting[16] are less than our instrument response, FWHM/2 from above has been reported instead (see Table 2 in main manuscript).

B-4) Evolution associated difference spectra (EADS)

Global sequential fitting of the type undertaken *via* Glotaran[16] in this study is described as:

$$\Psi(\lambda,t) = \sum_{i=1}^{n} c_i^{EADS}(t) EADS_i(\lambda) \quad (3)$$

where $\Psi(\lambda,t)$ is the data matrix to be fit for each probe wavelength λ and pump-probe time delay t, $c_i^{EADS}(t)$ is the exponentially decaying coefficient of the i-th component convolved with a Gaussian instrument response function, $EADS_i(\lambda)$ is the evolution associated difference spectrum for the i-th component. Glotaran therefore models the collected TAS as a superposition of n exponentially decaying spectral components.

EADS demonstrate the spectral profiles which decay with each lifetime assigned from global fitting. As such, they can aid in elucidating what ultrafast processes are being observed in collected TAS. EADS were obtained from global analysis of each sample (Figure S4).

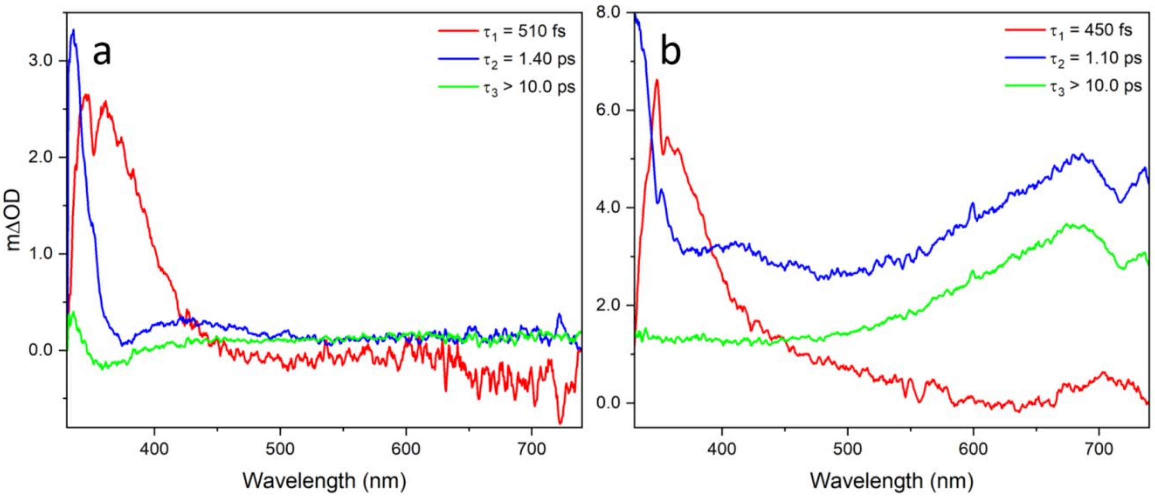

Figure S4. EADS extracted from global analysis of MyG at (a) pH 5.0 and (b) pH 1.0. The long-lived positive signal centred at ~690 nm in pH 1.0 is assigned to non-linear effects, confirmed by power dependence studies undertaken for this feature and the positive feature at ~340 nm.

In the global fitting analysis undertaken, a sub-100 fs lifetime was included to account for time-zero artefacts for both systems. This lifetime is not instructive in unravelling ultrafast photochemistry and (along with its respective EADS) was omitted due to its duration being far shorter than our instrument response FWHM.

B-5) Residuals from global analysis

Residuals were obtained from Glotaran[16] global fitting analysis of collected (non-chirp corrected) TEAS for each sample (Figure S5).

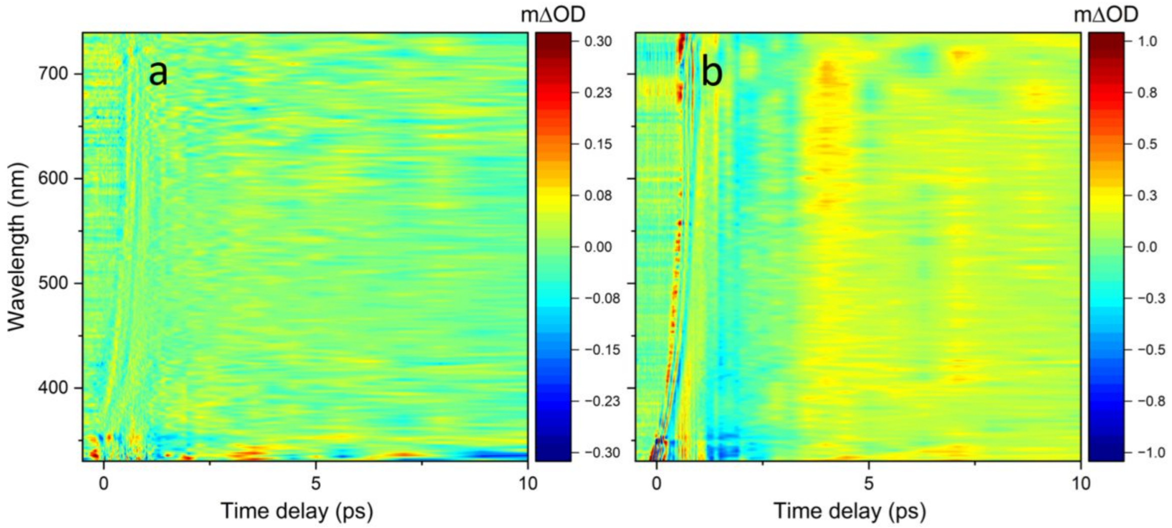

Figure S5. Residuals extracted from (non-chirp corrected) global analysis of MyG at (a) pH 5.0 and (b) pH 1.0.

B-6) Photostability of MyG solutions

MyG at pH 1.0 was found to degrade substantially in solution, even in the dark, generating, *via* hydrolysis, its known biosynthetic precursor 4-deoxygadusol (4-DG) with an absorption maximum at 268 nm.[17–19] We carried out a kinetic study (Figure S6) on the degradation of MyGH at pH 1.0, using respective absorption maxima for MyG and 4-DG to monitor the depletion of the former species and growth of the latter.

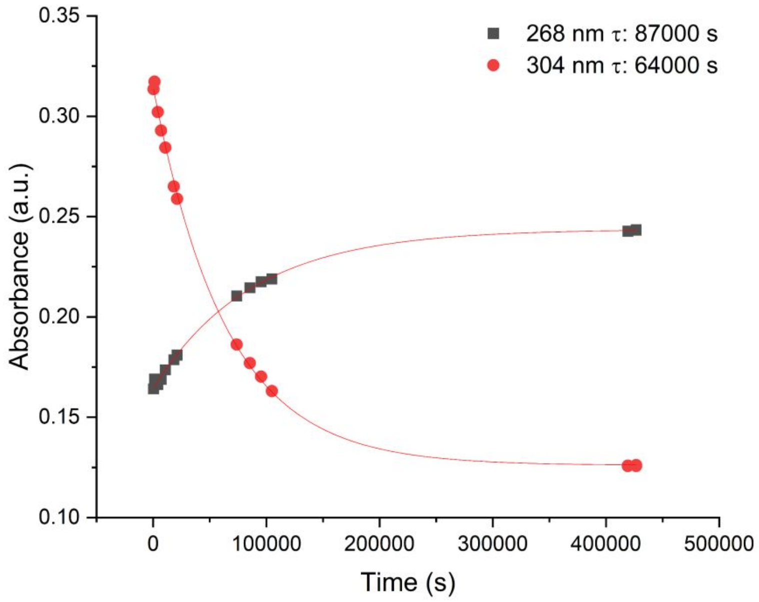

Figure S6. Absorption at 304 nm (red circles, corresponding to $MyGH^+$) and 268 nm (grey squares, corresponding to 4-DG) against time for MyG in pH 1.0 in the dark. Mono-exponential lifetimes for $MyGH^+$ decay and 4-DG growth respectively are also shown.

Likewise, MyG at pH 5.0 was found to degrade in solution, but only appreciably under a solar spectrum (Figure S7). This observation, although initially suggesting that MyG is not behaving as an effective UV filter under biological conditions, is qualified by the fact that, in Nature, the ensuing 4-DG photoproduct can be recycled in the microbial species to reform MyG.[19]

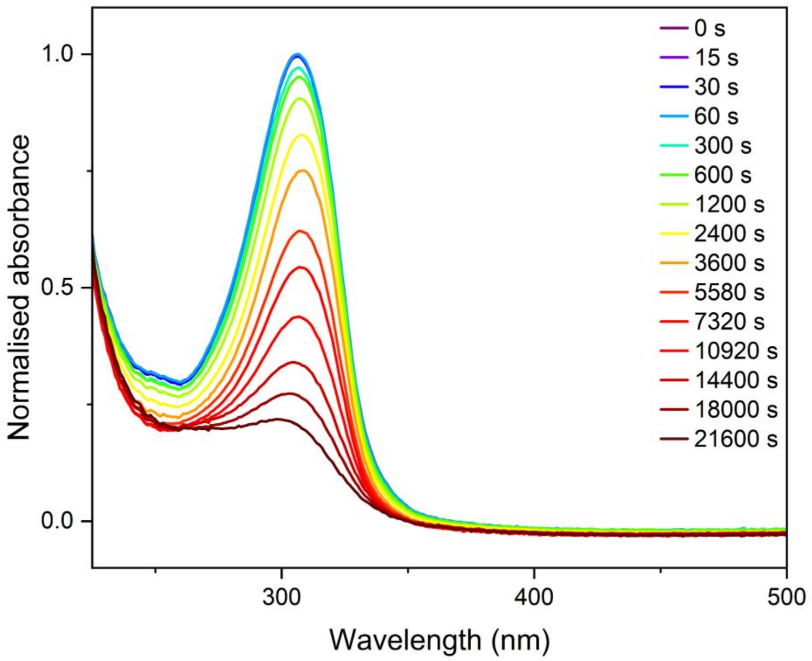

Figure S7. Absorption profile for MyG at pH 5.0 following irradiation with a solar simulator for given times. Irradiation was continued for 6 h to demonstrate the eventual growth of a feature at and below ~250 nm.

Section C

C-1) Computational details

The optimised geometry of protonated MyG at the ground state was determined at the RI-MP2 level of theory using the cc-pVDZ basis set.[20] The vertical excitation energies of the lowest excited singlet states and oscillator strengths for electronic transitions were computed based on the RI-ADC(2), TD-DFT/ωB97XD and multistate complete active space second-order perturbation (MS-CASPT2) theory.[21,22] In addition, the minimum energy geometries for conical intersections (CIs) have been determined based on a state-averaged complete active space self-consistent field (SA-CASSCF) theory.[23] We have determined the potential energy curves representing the deactivation mechanism of our system using a linear interpolation of internal coordinates (LIIC) at the MS-CASPT2/SA-CASSCF (6,6)/cc-pVDZ theoretical level. The active space in the SA-CASSCF calculations contains 6 electrons in 6 molecular orbitals (three occupied and three virtual molecular orbitals). This active space was previously used in determination of CIs for biomolecular systems as well as for neutral MyG in our previous work.[24–31]

The RI-MP2 and RI-ADC(2) calculations were carried out using the Turbomole program suite (V 6.3).[32,33] The DFT and TD-DFT calculations were employed using the Gaussian 16 program and SA-CASSCF/MS-CASPT2 computations were performed using OpenMolcas (V 18.09).[34–38]

Further, the UV-visible absorption spectrum and non-adiabatic dynamics simulation have been conducted based on TD-DFT using Newton-X (V 2.0) interfaced with Gaussian 16.[39] The validity of the selected model for determining excited state deactivation processes has been confirmed in different reports.[40–44] To perform nonadiabatic dynamics (NAD) simulations, initial conditions were computed for geometry and nuclear momenta sampled from a Wigner distribution based on the S_0 normal modes. Vertical excitation energies were computed at the ωB97XD/6-31G* level and these energies were obtained with δ = 0.05 eV Lorentzian line broadening for the two lowest singlet excited states. To investigate solvent influence on absorption spectra, the polarisable continuum model (PCM/water model) implemented in Gaussian 16, and the COSMO model implemented in Turbomole, have also been used.

Label	**1**	**2**	**3**	**4**	**5**
Geometry	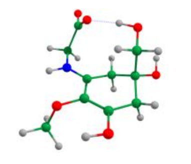	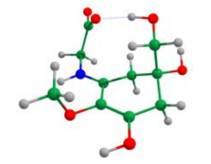	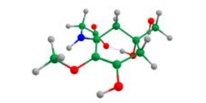	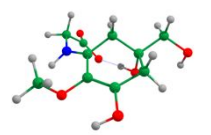	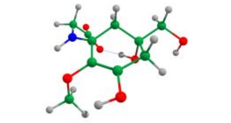
Relative Energy	0.00	0.96	1.93	4.82	5.80
**Label**	**6**	**7**	**8**	**9**	**10**
Geometry	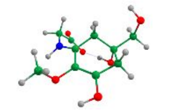	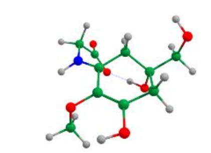	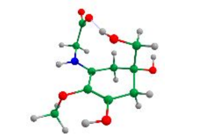	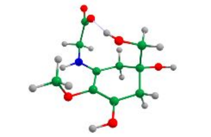	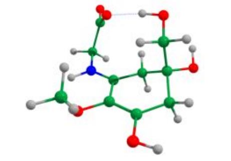
Relative Energy	8.70	9.65	9.7	10.6	11.6

Figure S8. Optimized structures (along with relative energy in kJ mol^{-1}) of the 10 most stable structures of zwitterionic MyG at the RI-MP2/cc-pVDZ level of theory in an implicit water solvent model.

C-2) Ground state optimised geometries

The conformational landscape of $MyG_{Zwitter}$ was determined based on a novel metadynamics (MTD) scheme for generating conformational ensembles, implemented in the Crest program (V. 2.12)[45], developed by Grimme and coworkers based on the metadynamics-driven search algorithm which utilises the GFN2-xTB (Geometries Frequencies Non-covalent interactions – extended Tight Binding, version 6.4.0) functional.[46] For conformational energies at a semiempirical level we used 30.0 kcalmol^{-1} as our energy window. Among the 330 obtained

conformers, we selected the 80 lowest lying structures ($\Delta E < 20$ kJmol^{-1}) and determined the minimum geometry and consequently the relative stabilities at the DFT/B3LYP/cc-pVDZ theoretical level in an implicit water solvent. Next, the 20 most stable structures were selected and geometrically optimized at the RI-MP2/cc-pVDZ theoretical level. We present the 10 lowest lying structures in Figure S8. As shown, structure **1** has been assigned as the most stable and we have selected this system for further calculations. To obtain the optimized structure for protonated MyG we used the already-optimized structure of the neutral analogue, based on a comprehensive conformational search performed in our previous work.[30]

As discussed above, there are roughly 30 conformers of MyG$_{zwitter}$ with internal energy of less than 20 kJ mol^{-1}, and conformer **1** has been proposed as the most stable. Regarding protonated MyG, according to our previous study, we have selected the most stable structure of neutral MyG and searched for the most stable isomer arising from its protonation.[31] The additional proton could be located at one of three different sites (C, O or N); hence, three protonated structural isomers were possible. We present the optimised structures of these possible protonated isomers in Figure S9. As shown, when the proton is located on O$_9$, the most stable protonated isomer (hereafter called MyGH$^+$) is obtained. As shown in Figure S9, this structure is quite similar to that of the most stable structure of MyG$_{zwitter}$. The second most stable isomer arises from protonation at C$_5$ in the carbon ring, 0.11 eV (10.50 kJmol^{-1}) less stable than MyGH$^+$; the third arises from protonation at N in the glycine side chain, 0.48 eV (46.31 kJmol^{-1}) less stable than MyGH$^+$. It is worthwhile noting that locating the additional proton over the O atom in the carboxylic group does not converge to a new protonated isomer, and instead converts to the MyNH$^+$ isomer following the MP2 geometry optimisation. As two distinct protonated isomers are >10 kJmol^{-1} less stable than MyGH$^+$, these would be substantially less populated at thermal equilibrium in our experiments, and so we neglect performing any further calculations on these.

Comparing the internal energy of the neutral MyG and that of optimised MyGH$^+$, we determine a proton affinity of -10.457 eV at the RI-MP2/cc-pVDZ level of theory. In water, the high proton affinity of MyG justifies the proton transfer from the solvent to form MyGH$^+$.

Further details of the xyz coordinates for the most stable isomers can be found at the end of this SI (Table S2-5).

MyNH$^+$ (0.48 eV)	MyCH$^+$ (0.11 eV)	MyGH$^+$ (0.00 eV)	MyGH$^+$·3H$_2$O	Zwitterion	MyGH$_{zwitter}$·3H$_2$O
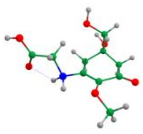	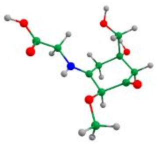	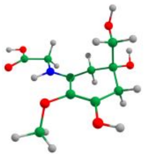	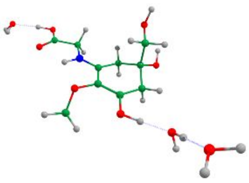	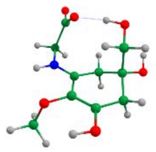	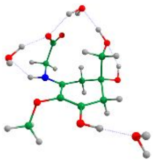

Figure S9. Optimised geometry of the three most stable structures of protonated MyG and the most stable structure of MyG$_{zwitter}$, determined at the RI-MP2/cc-pVDZ level of theory.

C-3) Electronic transition energies

Vertical transition energies and oscillator strengths of optimised MyGH$^+$, as well as MyG$_{zwitter}$, to the four lowest lying singlet excited states (S$_1$-S$_4$) have been determined and are presented in Table 1 (main text) and Table S1 respectively. We have employed different levels of theory: TD-DFT using the ωB97XD functional, RI-ADC(2), and MS-CASPT2.

Selected valence MO's of MyGH$^+$ are presented in Figure S10. According to our ADC(2) results, the S$_1$←S$_0$ electronic transition has been assigned as a $^1\pi\pi^*$ transition, originating from a LUMO←HOMO (85%) single electron transition. The second to fourth singlet electronic transitions have been assigned as optically dark $^1n\pi^*$ states. It has been predicted that the S$_2$←S$_0$ electronic transition arises from LUMO←HOMO-2 (92%), the S$_3$←S$_0$ transition arises from LUMO←HOMO-1, and the S$_4$←S$_0$ transition arises from LUMO←HOMO-3 and LUMO←HOMO-4.

We have also determined the lowest lying electronic transitions of $MyGH^+$ based on TD- ωB97XD and MS-CASPT2. The MS-CASPT2 results were obtained based on 6 electrons and 6 valence orbitals (6 occupied and 6 virtual) active space, involving 5 multistate. As shown in Table 1 (main text), the gas phase MS-CASPT2 results for the S_1 state (4.05 eV) agrees well with the ADC(2) level, but the TD-DFT/ωB97XD result of 4.318 eV is slightly overestimated.

According to our theoretical results the remaining three electronic transitions lie within 5.0-6.0 eV (at ADC(2) and TD-DFT level) and 5.40-7.0 eV (at the MS-CASPT2 level) above the S_0 ground state. Due to the 'dark' ($^1n\pi^*$) nature of these electronic transitions, their contribution to the UV-visible absorption spectrum of $MyGH^+$ is minimal. Our results concerning transition assignments based on TD-DFT and MS-CASPT2 confirm the $^1\pi\pi^*$ nature of the S_1 state as well as the $^1n\pi^*$ of the remaining three. Thus, the strong $S_1 \leftarrow S_0$ transition is imputed as responsible for the UV-visible absorption of protonated MyG.

In addition, we have determined the four lowest lying electronic transitions of $MyGH^+$ considering the effect of an implicit solvent based on COSMO (in Turbomole) and PCM models (in Gaussian and OpenMolcas, see Table 1 in main text). The $S_1 \leftarrow S_0$ ($^1\pi\pi^*$) electronic transition of $MyGH^+$ exhibits no alteration in the presence of solvent at the ADC(2) and TD-DFT levels, but the MS-CASPT2 result shows a slight blue shift (~0.30 eV). The same is also true for other electronic transitions but are less important in the UV-visible absorption of $MyGH^+$.

Concerning the $MyG_{zwitter}$, at the same theoretical levels discussed above for $MyGH^+$, we have determined the four lowest lying electronic singlet transitions, featuring an implicit water solvent model (see Table S1). Involving the solvent model is necessary for determination of physical properties of the zwitterion, as it provides a crucial stabilising factor. As shown in Table S1, the S_1-S_0 transition, owing to the largest oscillator strength (0.413 at the ADC(2) level), is responsible for the UV absorption of this system. We have determined a transition energy of 4.050 eV (at the ADC(2) theoretical level) approximately equal to that of its corresponding protonated analogue, 4.00 eV. The MS-CASPT2 results for this transition energy in $MyG_{zwitter}$ and $MyGH^+$ are in good agreement (4.30 and 4.35 eV respectively). Meanwhile, the TD-DFT results for the S_1-S_0 transition of zwitterion has been slightly overestimated (4.424 eV) compared to its protonated analogue (4.36). Nevertheless, all three theoretical levels confirm that the S_1-S_0 electronic transition in the zwitterion is close to that in the protonated case. We have also compared the theoretical results of these two systems with the experimental spectra recorded for two different solution environments (at pH 5.0 and 1.0 in Figure 3 in the main text and in Figure S11 below). The agreement between our theoretical results and experiments indicates two important points:

(i) It shows how reliable our theoretical models are for describing photophysics of these systems. (ii) The similarity in electronic transitions and electronic structures suggests that photophysics of the zwitterion and its protonated analogue should be similar. For this reason and due to the high computational costs, we ignored further calculations on the zwitterion and instead have focused on the protonated system.

Table S2. Vertical transition energies and oscillator strengths for the most stable conformer of $MyG_{zwitter}$, in the water solvent phase at different theoretical levels with cc-pVDZ basis set. The values in parentheses represent the oscillator strengths. The MS-CASPT2 results have been obtained based on 4 electrons and 4 orbitals as the active space and 5 multistate.

Excited state	Transition Energy/eV		
	ADC (2)	TD-ωB97XD	MS-CASPT2
	Cosmo/water	PCM/water	microhydrated
S_1	4.05<br>(0.4128)	4.42<br>(0.4519)	3.98<br>(0.5861)
S_2	4.01<br>(0.0061)	4.69<br>(0.0053)	4.77<br>(0.0310)
S_3	4.56<br>(0.0202)	5.08<br>(0.0075)	5.18<br>(0.1377)
S_4	4.77<br>(0.0097)	5.17<br>(0.0015)	6.09<br>(0.0288)

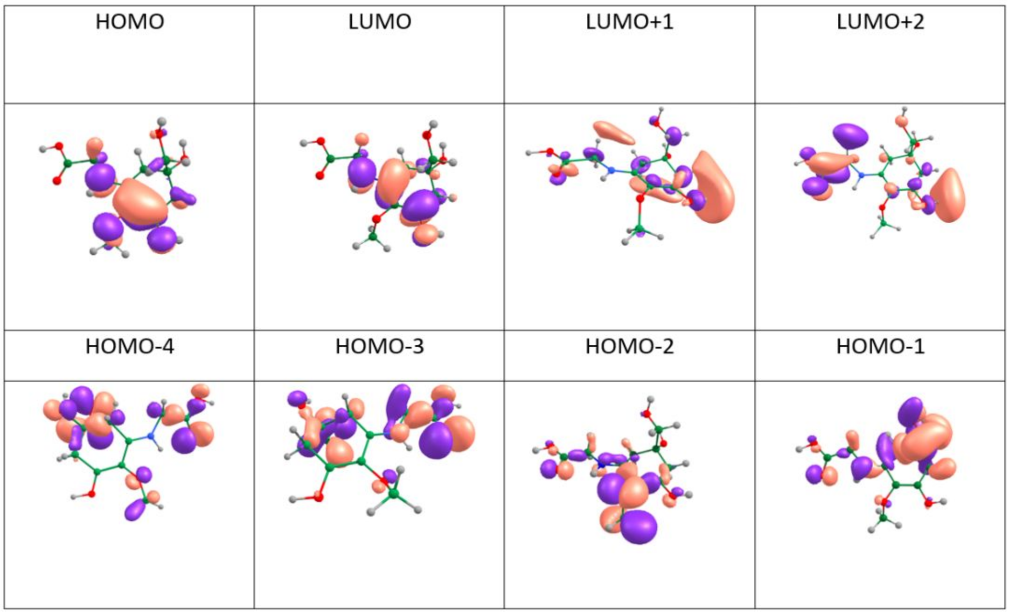

Figure S10. Selected valence molecular orbitals of MyGH+, determined at the SCF/cc-pVDZ level of theory, which play a prominent role in the lowest lying electronic transitions.

In addition to using the implicit solvation model, we have considered a 'micro-hydrated' model to mimic the effect of explicit solvent around our systems. Following consideration of different locations for 3 additional water molecules, and determination of the optimized ground state structures of several candidates at the MP2/aug-cc-pVDZ, the most stable micro—hydrated structures were found (these structures are presented in Figure S9). Electronic transition energies were then determined at the ADC(2), TD-DFT and MS-CASPT2 theory levels. The S_1-S_4 electronic transitions calculated by the ADC(2) and TD-DFT computational methods for this micro-hydrated model are seen as similar to those of the implicit solvent model; however, the MS-CASPT2 results exhibit a 0.3-0.4 eV red shift once the explicit solvent effect (i.e., addition of 3 water molecules) has been considered. For this reason, in Table 1 (see main text) and Table S1, only the MS-CASPT2 results for the micro-hydrated model are presented. The results for other methods have been presented using the implicit solvent model.

C-4) Conical intersections and potential energy profiles

We located possible CIs for $MyGH^+$ based on the SA-CASSCF (6,6)/cc-pVDZ theoretical model implemented in OpenMolcas program. Two optimised CIs were determined (Figure S12). The active space in the SA-CASSCF calculations contains 6 electrons in 6 MO's (three occupied and three virtual MO's, see Figure S10).

In CI_1 the $MyGH^+$ shows strong deformation in the C_3-C_6 region. Ring puckering from C_6 is accompanied by strong movement of the glycine side chain upward out of the ring. In parallel to C_6, the C_3 moiety is moved upward; the net result of these motions is the formation of a 'boat' shape for $MyGH^+$ at the optimised CI_1. It is worth noting that slight ring puckering from C_6-C_3 is predicted for the optimised S_1 structure of $MyGH^+$ and the CI_1 is located somewhere after the minimum geometry on the S_1 excited state potential energy surface, where significant deformation of the six membered ring from C_3-C_6 regions is predicted.

In CI_2, the 'boat' structure is obtained by deformation of the six-membered ring of $MyGH^+$ from the C_2-C_4 region. Accordingly, the glycine side chain moves in the opposite direction compared to CI_1. The Cartesian coordinates of the optimised CI structures can be found at the end of this SI.

As locating CIs does not confirm the mechanism of nonradiative deactivation, we have determined potential energy profiles for the ground and S_1 electronic states for $MyGH^+$ based on the linear interpolation of internal coordinates (LIIC), connecting the Franck-Condon (FC) region to CIs, based on the MS-CASPT2/SA-CASSCFT/cc-pVDZ theoretical level. As above, we selected 6 electrons in 6 MOs as the active space. Most of these results are already presented in the main text (see Figure 4c) and the complementary sections are shown in Figure S14.

C-5) Non-adiabatic dynamics (NAD) simulations

We have employed nonadiabatic dynamics (NAD) simulations based on the TD-DFT theoretical model on the most stable isomer of $MyGH^+$. In line with our previous study on neutral MyG, we have here used a nonadiabatic surface-hopping dynamics simulation from the S_1 ($^1\pi\pi^*$) excited state at the TD-ωB97xD/6-31G* level of theory, with water as an implicit solvent. The UV-visible absorption spectrum of $MyGH^+$, shown in Figure S15, was determined based on the nuclear ensemble approach.[47] The velocity Verlet algorithm with a nuclear time step of 0.5 fs was used for the integration of Newtonian dynamical equations.[48] The NAD simulation was initialised from the S_1 ($^1\pi\pi^*$) excited state, using the decoherence-corrected fewest switches surface-hopping (DC-FSSH) approach.[49,50] 57 trajectories from the optically bright S_1 state were considered. Initial conditions for the dynamics were sampled within the 4.2 ± 0.10 eV spectral window highlighted in Figure S15. Population in a trajectory was considered to have returned to the S_0 when the S_1-S_0 energy gap became < 0.20 eV. Surface-hopping dynamics were simulated for a maximum of 800 fs. 100% of S_1 population was found to transfer to S_0 within 400-600 fs. The deactivation mechanism was therefore found to consist of driving S_1 population across the excited state potential energy surface (see Figure S16), before reaching a CI. The net effect is the efficient conversion of absorbed UV light (4.20 eV) into heat *via* vibrational redistribution. These predicted dynamics are in good agreement with our previously discussed *ab initio* results.

We have also acknowledged geometry alterations undergone during trajectories. It was revealed that 42 of 57 trajectories relaxed by ring puckering from the C_6-C_3 region; although, in a few cases, ring deformation occurs individually from C_3 *or* C_6, both atoms are generally involved in deformation, yielding a 'boat' structure at the CI_1. The remaining 15 trajectories relax by ring deformation from C_2, C_4, or both.

We present also the time evolution of the nonadiabatic population of the ground and first two excited states from the NAD simulation of $MyGH^+$ (Figures S16-18). Using a sigmoid function[51] for curve fitting as an approximate guide, we determine the S_1 excited state lifetime as 500 fs which is in excellent agreement with our experimental result (τ_1 = 510 fs at pH = 1.0). From this, we conclude that photoexcitation of $MyGH^+$ to S_1 can relax to the S_0 ground state in approximately ~0.5 ps. This ultra-short lifetime of the excited state strongly supports the high photostability of $MyGH^+$ even in an acidic environment.

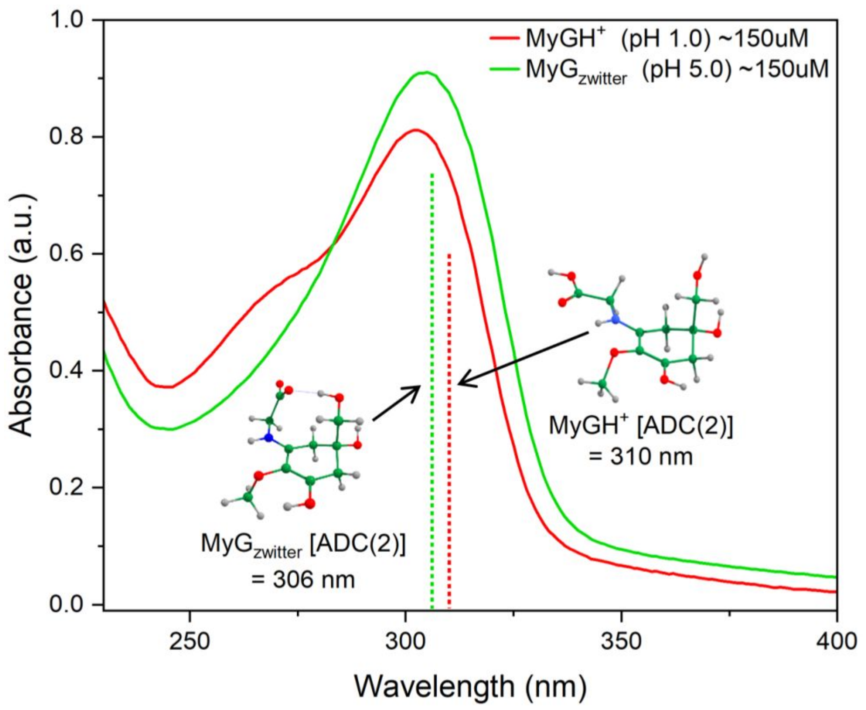

Figure S11. Comparison between experimental UV-visible absorption spectra of protonated (pH = 1.0) and zwitterionic MyG (pH = 5.0) (red and green lines respectively). The vertical lines represent theoretical results for the $S_1 \leftarrow S_0$ electronic transitions of protonated and zwitterionic structures of MyG, determined at the ADC(2)/cc-pVDZ theoretical level in an implicit water solvent model. The insets are optimized geometries of protonated and zwitterionic MyG.

a) CI_1	b) CI_2
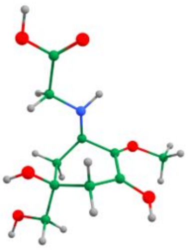	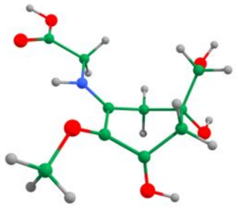

Figure S12. Optimized geometries of CI_{1-2} (S_1/S_0) determined at the SA-CASSCF (6,6)/cc-pVDZ theoretical level.

	HOMO	LUMO
$MyGH^+$	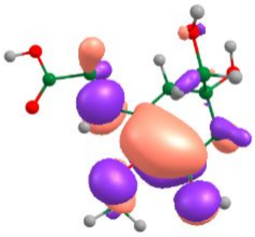	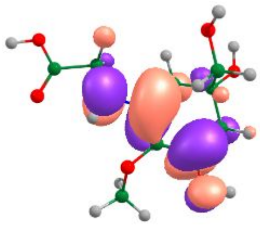
$MyG_{zwitter}$	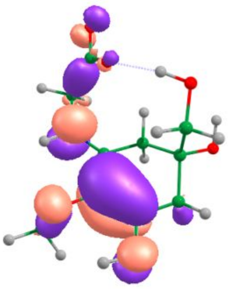	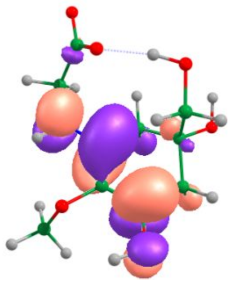

Figure S13. Comparison of the HOMO/LUMO electronic distributions for $MyGH^+$ and $MyG_{zwitter}$.

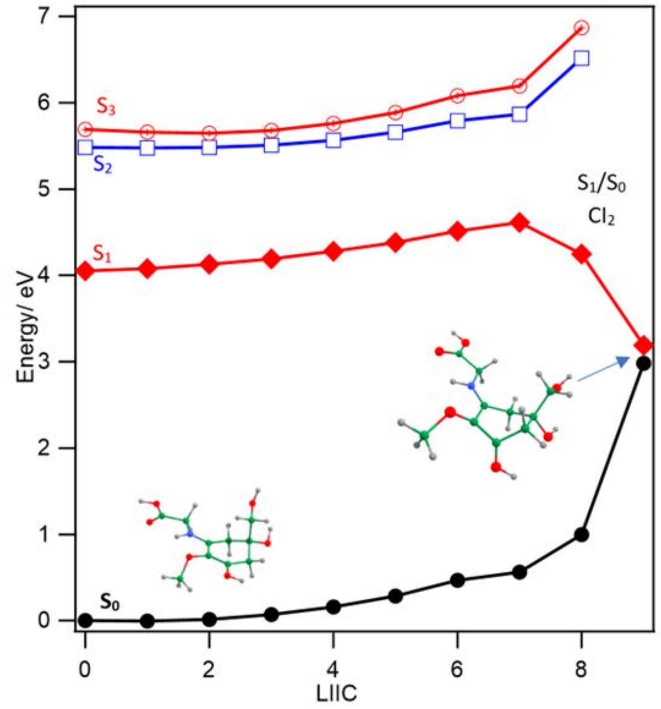

Figure S14. Potential energy profile of the ground (S_0) and excited (S_1-S_3) states of $MyGH^+$ calculated at the MS-CASPT2/CASSCF(6,6)/cc-pVDZ level of theory along the LIIC reaction path. The left inset structure represents the optimised geometry of $MyGH^+$ in the FC region and the right structure the relevant optimised CI_2 structure.

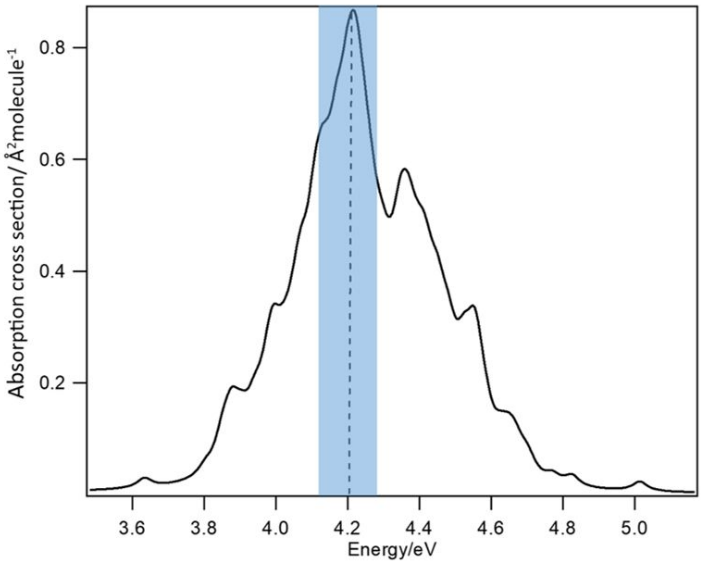

Figure S15. UV-visible absorption spectrum for $MyGH^+$ simulated at the TD-ωB97XD/6-31G* method, based on the $S_1 \leftarrow S_0$ electronic transition, in a PCM/ethanol implicit solvent with 500 points. The dashed line represents the maximum of absorption cross-section (at 4.20 eV) and the shaded area indicates the spectral window from where the initial conditions for the dynamics were sampled.

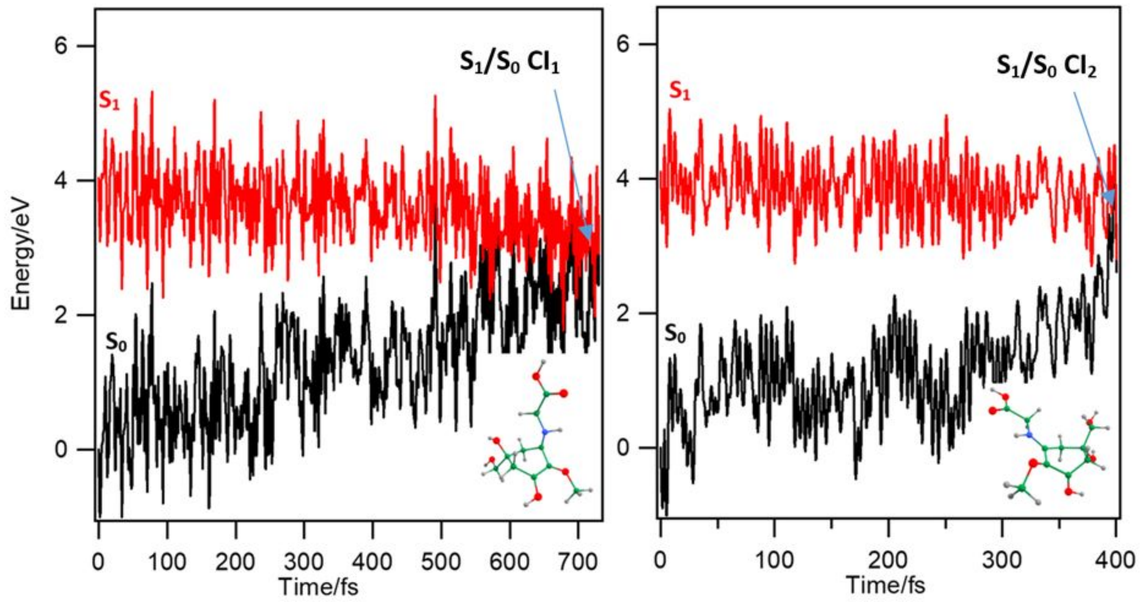

Figure S16. Energy profiles of a selected NAD trajectory for $MyGH^+$, (left) based on the first deactivation path (ring puckering from C_3-C_6) and (right) the second deactivation path (ring puckering from C_2-C_4). The black and red curves respectively indicate the ground (S_0) and S_1 states. Insets: corresponding geometry of trajectory ending at S_1/S_0 curve crossing (i.e., CIs).

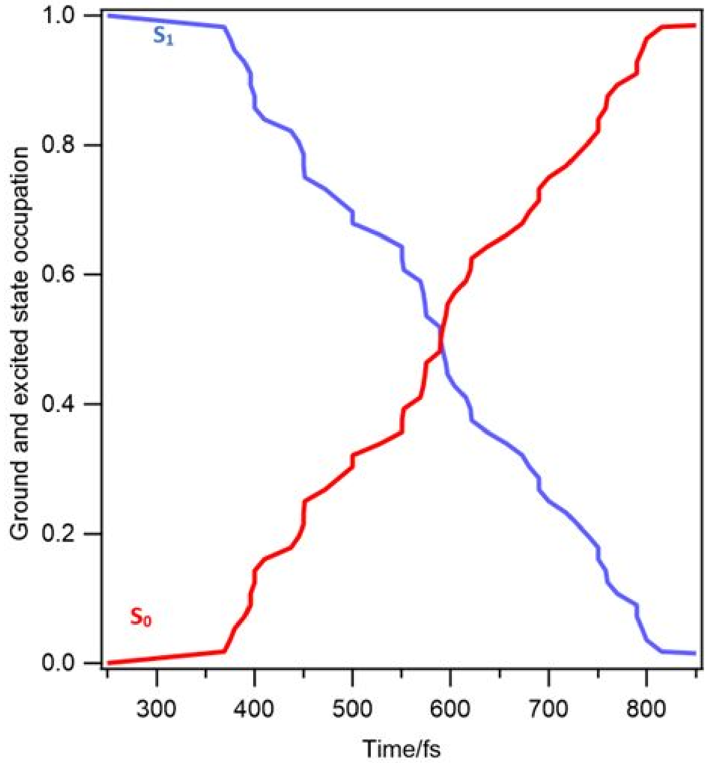

Figure S17. Time evolution of the population of the ground (S_0) (red) and first excited state (S_1) (blue) from NAD simulation for $MyGH^+$.

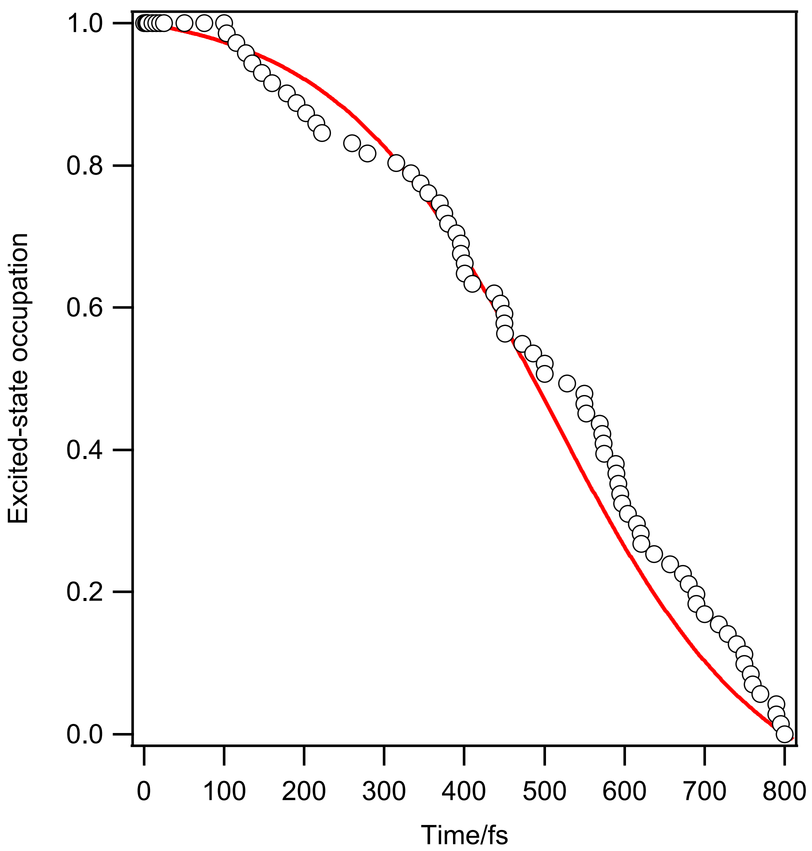

Figure S18. Excited state occupation (fraction of trajectories in excited states) as a function of time for $MyGH^+$. The red curve represents the fitted sigmoid function (see SI section C-5 and the publication by Toldo *et al*[51] for discussion), the extracted lifetime from which is 500 ± 5 fs.

C-6) Geometry propagations following nonradiative relaxation of $MyGH^+$

To clarify the deactivation mechanism for the S_1 excited state of $MyGH^+$, we have analysed all (57) trajectories describing relaxation of the system. Cremer-Pople parameters[52] were computed to analyse the out-of-plane ring distortion in the simulations using the PLATON[53] program. The distribution of trajectories in the space of the Cremer-Pople parameters (Q, θ, and ϕ) is shown in Figure S19. The trend of ring-deformation is described by the parameters θ and ϕ. As shown in Figure S19, the excited S_1 state $MyGH^+$ propagates in time *via* one of two types of ring deformations. The first branch corresponds to the C_3-C_6 twisting coordinates (θ~85°, ϕ ~120°) and the second group results from out-of-plane deformation of the ring at the C_2-C_4 sites (θ~100°, ϕ ~220°). The distribution of deactivation paths shows that C_3-C_6 plays a prominent role in internal conversion.

Moreover, the time evolution of the four dihedrals angles (α-ω) responsible for the two deactivation pathways (i.e., C_3-C_6 and C_2-C_4 twisting) ending at the $^1\pi\pi^*/S_0$ intersection is represented in Figure S20. These results have been extracted from the trajectory files using a homemade Python code. The positive values obtained are due to out-of-plane bending of the atoms towards the top of the cyclohexenone ring. Initially, each of the abovementioned dihedral angles is around 0° (indicating a flat cyclic structure), which increases to 20° or 40° degrees at the crossing points.

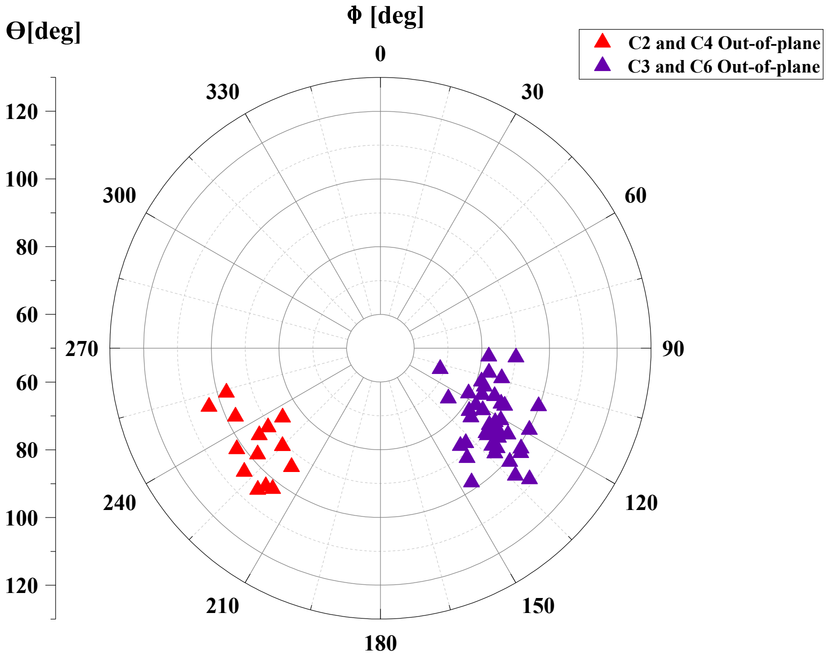

Figure S19. Polar plot showing the distribution of Cremer-Pople parameters θ and φ at the S_1/S_0 crossing geometry of each trajectory.

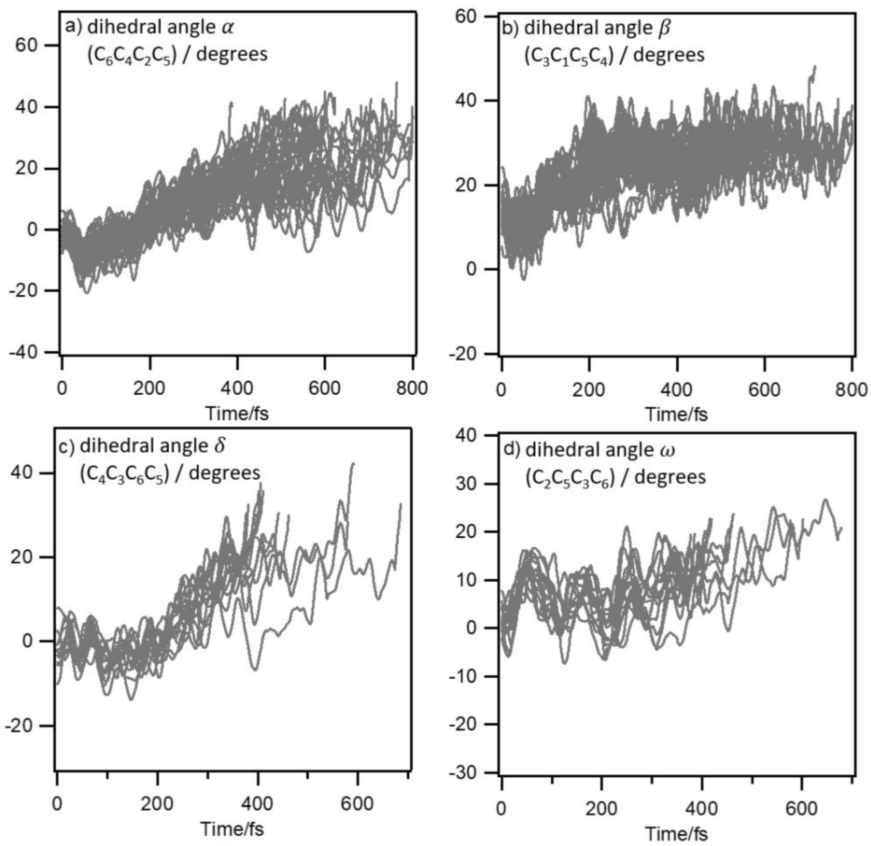

Figure S20. Time evolution of the dihedral angles of α ($C_6C_4C_2C_5$), β ($C_3C_1C_5C_4$), δ ($C_4C_3C_6C_5$) and ω ($C_2C_5C_3C_6$) responsible for ring twisting at the C_6-C_3 and C_2-C_4 region of $MyGH^+$ following trajectory simulation.

Table S2. xyz coordinates of the ground state optimized geometry (S_0) for the most stable isomer of $MyGH^+$ calculated at the MP2/cc-pVDZ level of theory.

C	-2.06721	-1.11147	-0.36711
C	-0.59747	-1.03021	-0.83107
C	-2.75024	0.23672	-0.60706
C	-1.95622	1.39947	-0.08683
C	-0.58443	1.35413	0.07626
C	0.11287	0.14202	-0.23255
H	-0.08630	-1.97296	-0.58396
H	-3.75118	0.23507	-0.13723
O	-2.57683	2.54267	0.21317
O	0.16354	2.37307	0.60014
N	1.40377	0.11781	0.01695
O	-2.76098	-2.05117	-1.15942
C	-2.14219	-1.54222	1.10226
O	-1.51764	-2.82169	1.13532
C	0.26301	3.53206	-0.26473
H	-1.76247	-3.25485	1.96552
H	-0.59847	-0.91797	-1.93194
H	-2.90921	0.34868	-1.69696
H	1.82076	0.96947	0.42181
H	-2.51820	-2.91926	-0.79188
H	-1.63578	-0.81739	1.76815
H	-3.21017	-1.60041	1.38357
H	0.99147	4.19725	0.21607
H	0.62486	3.23564	-1.26401
H	-0.71003	4.03752	-0.34663
C	2.32887	-0.98237	-0.20981
C	3.71601	-0.46283	0.15400
O	3.91421	0.66222	0.56526
O	4.63289	-1.41211	-0.04365
H	5.49695	-1.03187	0.20699
H	2.31932	-1.30779	-1.26281
H	2.08443	-1.85101	0.42479
H	-3.53192	2.44278	0.06039

Table S3. xyz coordinates of the optimised CI_1 geometry for $MyGH^+$, calculated at the SA-CASSCF(6,6)/6-31G* level of theory.

C	-1.42854134	-1.07776668	-0.12980623
C	-0.41590003	-0.87723736	1.02748438
C	-1.58403246	0.17599860	-1.04790959
C	-1.33725484	1.47748442	-0.35121032
C	-0.18404748	1.58230039	0.56378540
C	0.55077586	0.25635499	0.73782916
H	-0.97013946	-0.64900636	1.93988335
H	-2.52765536	0.16869929	-1.56296252
O	-2.12080440	2.49801431	-0.47775466
O	0.17244336	2.56642401	1.25352477
N	1.47219153	0.16290289	-0.27403964
O	-0.96535256	-2.05752756	-1.00524607
C	-2.78704119	-1.51403110	0.40709809
O	-2.58502821	-2.68263432	1.13832402
C	-0.41534707	3.89557964	1.25909176
H	-3.39475055	-3.03873895	1.46529759
H	0.09733867	-1.80865751	1.25447244
H	-0.80036017	0.06687071	-1.81184035
H	2.11142223	0.94070270	-0.35215261
H	-1.14517996	-2.89972862	-0.59920384
H	-3.27110331	-0.77101940	1.08073729
H	-3.53848533	-1.67687251	-0.37837694
H	0.19993201	4.51429970	1.89123943
H	-0.41128095	4.21306789	0.21759289
H	-1.42485054	3.80902000	1.64174247
C	2.17300154	-1.08284225	-0.48578450
C	3.33029357	-0.85263014	-1.41552244
O	3.72641711	0.22095523	-1.74954285
O	3.86465964	-1.99557476	-1.81578398
H	4.61745800	-1.81759193	-2.36088967
H	1.49847234	-1.83397060	-0.92735633
H	2.56130088	-1.51348516	0.42566419
H	-2.86251153	2.33962044	-1.06536472

Table S4. xyz coordinates of the optimised CI_2 geometry for $MyGH^+$, calculated at the SA-CASSCF(6,6)/6-31G* level of theory.

C	-2.06317520	-0.89933419	-0.06125873
C	-0.76450454	-0.61187653	-0.89680211
C	-2.58288230	0.35394972	0.71989350
C	-2.03829691	1.49185076	-0.06131295
C	-0.59317331	1.66181503	0.05447371
C	0.08964344	0.50783151	-0.33464146
H	-0.20287173	-1.53425573	-1.00240416
H	-2.17872981	0.38585877	1.73024189
O	-2.61124847	1.88210970	-1.15781003
O	-0.01993289	2.51043442	0.92495466
N	1.37977111	0.37195458	-0.08699204
O	-3.11446896	-1.20857290	-0.98086704
C	-1.86893158	-2.06688917	0.89923501
O	-1.58170253	-3.18422167	0.10935828
C	-0.25042496	3.90256567	0.72946060
H	-1.65137776	-3.98171516	0.61437875
H	-1.08728748	-0.32114246	-1.89445204
H	-3.66972997	0.30929072	0.75915093
H	1.85547988	1.15728415	0.32706854
H	-2.97268805	-2.09426267	-1.29720636
H	-1.05623697	-1.84897929	1.60008694
H	-2.78895740	-2.20844398	1.47112490
H	0.31257344	4.41193156	1.50437771
H	0.10177003	4.21104265	-0.25451296
H	-1.30966550	4.13755014	0.83494565
C	2.21735544	-0.78017661	-0.34902663
C	3.63170188	-0.43398596	0.06031881
O	3.93932806	0.61312098	0.51695854
O	4.44361283	-1.43994722	-0.15847060
H	5.32921955	-1.20163098	0.10263428
H	2.21803996	-1.04343420	-1.40575135
H	1.89854701	-1.65377445	0.21832520
H	-3.47333630	1.49286279	-1.28128942

Table S5. xyz coordinates of the optimised S_1 geometry for $MyGH^+$, calculated at the ADC(2)/cc-pVDZ level of theory.

C	-1.46014	-1.06264	-0.13740
C	-0.45154	-0.83916	1.03710
C	-1.64282	0.23523	-0.97002
C	-1.39364	1.50474	-0.21769
C	-0.21905	1.56181	0.58495
C	0.51466	0.26777	0.72723
H	-1.00578	-0.57145	1.95589
H	-2.63452	0.26131	-1.45521
O	-2.21005	2.54999	-0.29916
O	0.25406	2.59601	1.22629
N	1.40901	0.09299	-0.33958
O	-0.96094	-2.00834	-1.07108
C	-2.79873	-1.56893	0.40344
O	-2.47959	-2.79664	1.03714
C	-0.33887	3.93554	1.14988
H	-3.30437	-3.24867	1.26471
H	0.05314	-1.79652	1.24207
H	-0.87753	0.17254	-1.76675
H	2.02808	0.89113	-0.52696
H	-1.12713	-2.87299	-0.65161
H	-3.23756	-0.83086	1.10582
H	-3.49381	-1.71300	-0.44692
H	0.35393	4.56047	1.72220
H	-0.39099	4.24793	0.09893
H	-1.33464	3.91253	1.61007
C	2.14857	-1.15383	-0.46603
C	3.34428	-0.88406	-1.36436
O	3.70955	0.23048	-1.69045
O	3.94388	-2.02913	-1.72059
H	4.71714	-1.77767	-2.25978
H	1.51550	-1.93973	-0.90547
H	2.53368	-1.52053	0.50648
H	-2.95774	2.33266	-0.89012

References

(1) Gomez-Escribano, J. P.; Bibb, M. J. Engineering Streptomyces Coelicolor for Heterologous Expression of Secondary Metabolite Gene Clusters. *Microb. Biotechnol.* **2011**, *4*, 207–215.

(2) Baltz, R. H. Streptomyces and Saccharopolyspora Hosts for Heterologous Expression of Secondary Metabolite Gene Clusters. *J. Ind. Microbiol. Biotechnol.* **2010**, *37*, 759–772.

(3) Baltz, R. H.; Matsushima, P. Protoplast Fusion in Streptomyces: Conditions for Efficient Genetic Recombination and Cell Regeneration. *J. Gen. Microbiol.* **1981**, *127*, 137–146.

(4) Chater, K. F.; Wilde, L. C. Streptomyces Albus G Mutants Defective in the SalGI Restriction-Modification System. *J. Gen. Microbiol.* **1980**, *116*, 323–334.

(5) Kallifidas, D.; Jiang, G.; Ding, Y.; Luesch, H. Rational Engineering of Streptomyces Albus J1074 for the Overexpression of Secondary Metabolite Gene Clusters. *Microb. Cell Fact.* **2018**, *17*, 1–14.

(6) Lopatniuk, M.; Myronovskyi, M.; Nottebrock, A.; Busche, T.; Kalinowski, J.; Ostash, B.; Fedorenko, V.; Luzhetskyy, A. Effect of “Ribosome Engineering” on the Transcription Level and Production of S. Albus Indigenous Secondary Metabolites. Appl. *Microbiol. Biotechnol.* **2019**, *103*, 7097–7110.

(7) Clifton, K. P.; Jones, E. M.; Paudel, S.; Marken, J. P.; Monette, C. E.; Halleran, A. D.; Epp, L.; Saha, M. S. The Genetic Insulator RiboJ Increases Expression of Insulated Genes. *J. Biol. Eng.* **2018**, *12*.

(8) Lou, C.; Stanton, B.; Chen, Y. J.; Munsky, B.; Voigt, C. A. Ribozyme-Based Insulator Parts Buffer Synthetic Circuits from Genetic Context. *Nat. Biotechnol.* **2012**, *30*, 1137–1142.

(9) Bai, C.; Zhang, Y.; Zhao, X.; Hu, Y.; Xiang, S.; Miao, J.; Lou, C.; Zhang, L.; Demain, A. L. Exploiting a Precise Design of Universal Synthetic Modular Regulatory Elements to Unlock the Microbial Natural Products in Streptomyces. *Proc. Natl. Acad. Sci. U.S.A.* **2015**, *112*, 12181–12186.

(10) Osborn, A. R.; Mahmud, T. Interkingdom Genetic Mix-and-Match to Produce Novel Sunscreens. *ACS Synth. Biol.* **2019**, *8*, 2464–2471.

(11) Matsuyama, K.; Matsumoto, J.; Yamamoto, S.; Nagasaki, K.; Inoue, Y.; Nishijima, M.; Mori, T. PH-Independent Charge Resonance Mechanism for UV Protective Functions of Shinorine and Related Mycosporine-like Amino Acids. *J. Phys. Chem. A* **2015**, *119*, 12722–12729.

(12) Baker, L. A.; Greenough, S. E.; Stavros, V. G. A Perspective on the Ultrafast Photochemistry of Solution-Phase Sunscreen Molecules. *J. Phys. Chem. Lett.* **2016**, *7*, 4655–4665.

(13) Berera, R.; van Grondelle, R.; Kennis, J. T. M. Ultrafast Transient Absorption Spectroscopy: Principles and Application to Photosynthetic Systems. *Photosynth. Res.* **2009**, *101*, 105–118.

(14) Kovalenko, S. A.; Dobryakov, A. L.; Ruthmann, J.; Ernsting, N. P. Femtosecond Spectroscopy of Condensed Phases with Chirped Supercontinuum Probing. *Phys. Rev. A* **1999**, *59*, 2369-2384.

(15) Lorenc, M.; Ziolek, M.; Naskrecki, R.; Karolczak, J.; Kubicki, J.; Maciejewski, A. Artifacts in Femtosecond Transient Absorption Spectroscopy. *Appl. Phys. B* **2002**, *74*, 19–27.

(16) Snellenburg, J. J.; Laptenok, S.; Seger, R.; Mullen, K. M.; van Stokkum, I. H. M. Glotaran: A Java-Based Graphical User Interface for the R Package TIMP. *J. Stat. Softw.* **2012**, *49*, 1–22.

(17) Rosic, N. N. Mycosporine-like Amino Acids: Making the Foundation for Organic Personalised Sunscreens. *Mar. Drugs* **2019**, *17*.

(18) Ito, S.; Hirata, Y. Isolation and Structure of a Mycosporine from the Zoanthid Palythoa Tuberculosa. *Tetrahedron Lett.* **1977**, *18*, 2429–2430.

(19) Wada, N.; Sakamoto, T.; Matsugo, S. Mycosporine-Like Amino Acids and Their Derivatives as Natural Antioxidants. *Antioxidants* **2015**, *4*, 603.

(20) Dunning, T. H. Gaussian Basis Sets for Use in Correlated Molecular Calculations. I. The Atoms Boron through Neon and Hydrogen. *J. Chem. Phys.* **1989**, *90*, 1007–1023.

(21) Chai, J. Da; Head-Gordon, M. Long-Range Corrected Hybrid Density Functionals with Damped Atom–Atom Dispersion Corrections. *Phys. Chem. Chem. Phys.* **2008**, *10*, 6615–6620.

(22) Finley, J.; Malmqvist, P. Å.; Roos, B. O.; Serrano-Andrés, L. The Multi-State CASPT2 Method. *Chem. Phys. Lett.* **1998**, *288*, 299–306.

(23) Malmqvist, P. Å.; Roos, B. O. The CASSCF State Interaction Method. *Chem. Phys. Lett.* **1989**, *155*, 189–194.

(24) Perun, S.; Sobolewski, A. L.; Domcke, W. Conical Intersections in Thymine. *J. Phys. Chem. A* **2006**, *110*, 13238–13244.

(25) Perun, S.; Sobolewski, A. L.; Domcke, W. Ab Initio Studies on the Radiationless Decay Mechanisms of the Lowest Excited Singlet States of 9H-Adenine. *J. Am. Chem. Soc.* **2005**, *127*, 6257–6265.

(26) Yamazaki, S.; Sobolewski, A. L.; Domcke, W. Photophysics of Xanthine: Computational Study of the Radiationless Decay Mechanisms. *Phys. Chem. Chem. Phys.* **2009**, *11*, 10165–10174.

(27) Yamazaki, S.; Domcke, W.; Sobolewski, A. L. Nonradiative Decay Mechanisms of the Biologically Relevant Tautomer of Guanine. *J. Phys. Chem. A* **2008**, *112*, 11965–11968.

(28) Delchev, V. B.; Sobolewski, A. L.; Domcke, W. Comparison of the Non-Radiative Decay Mechanisms of 4-Pyrimidinone and Uracil: An Ab Initio Study. *Phys. Chem. Chem. Phys.* **2010**, *12*, 5007–5015.

(29) Abedini, F.; Omidyan, R.; Salehi, M. Theoretical Insights on Nonradiative Deactivation Mechanisms of Protonated Xanthine. *J. Photochem. Photobiol., A* **2019**, *385*, 112067.

(30) Omidyan, R.; Abedini, F.; Shahrokh, L.; Azimi, G. Excited State Deactivation Mechanism in Protonated Uracil: New Insights from Theoretical Studies. *J. Phys. Chem. A* **2020**, *124*, 5089–5097.

(31) Omidyan, R.; Shahrokh, L.; Whittock, A. L.; Stavros, V. G. Theoretical Insights into the Ultrafast Deactivation Mechanism and Photostability of a Natural Sunscreen System: Mycosporine Glycine. *J. Phys. Chem. A* **2023**, *127*, 4880–4887.

(32) TURBOMOLE V6.3, a Development of University of Karlsruhe and Forschungszentrum Karlsruhe GmbH, 1989-2007, TURBOMOLE GmbH, since 2007; Available from Http://Www.Turbomole.Com.

(33) Ahlrichs, R.; Bär, M.; Häser, M.; Horn, H.; Kölmel, C. Electronic Structure Calculations on Workstation Computers: The Program System Turbomole. *Chem. Phys. Lett.* **1989**, *162*, 165–169.

(34) Frisch, M. J.; Trucks, G. W.; Schlegel, H. B.; Scuseria, G. E.; Robb, M. A.; Cheeseman, J. R.; Scalmani, G.; Barone, V.; Petersson, G. A.; Nakatsuji, H.; Li, X.; Caricato, M.; Marenich, A. V.; Bloino, J.; Janesko, B. G.; Gomperts, R.; Mennucci, B.; Hratchian, H. P.; Ortiz, J. V.; Izmaylov, A. F.; Sonnenberg, J. L.; Williams-Young, D.; Ding, F.; Lipparini, F.; Egidi, F.; Goings, J.; Peng, B.; Petrone, A.; Henderson, T.; Ranasinghe, D.; Zakrzewski, V. G.; Gao, J.; Rega, N.; Zheng, G.; Liang, W.; Hada, M.; Ehara, M.; Toyota, K.; Fukuda, R.; Hasegawa, J.; Ishida, M.; Nakajima, T.; Honda, Y.; Kitao, O.; Nakai, H.; Vreven, T.; Throssell, K.; Montgomery, J. A., Jr.; Peralta, J. E.; Ogliaro, F.; Bearpark, M. J.; Heyd, J. J.; Brothers, E. N.; Kudin, K. N.; Staroverov, V. N.; Keith, T. A.; Kobayashi, R.; Normand, J.; Raghavachari, K.; Rendell, A. P.; Burant, J. C.; Iyengar, S. S.; Tomasi, J.; Cossi, M.; Millam, J. M.; Klene, M.; Adamo, C.; Cammi, R.; Ochterski, J. W.; Martin, R. L.; Morokuma, K.; Farkas, O.; Foresman, J. B.; Fox, D. J. Gaussian 16, Revision A3. 2016.

(35) Celani, P.; Werner, H. J. Multireference Perturbation Theory for Large Restricted and Selected Active Space Reference Wave Functions. *J. Chem. Phys.* **2000**, *112*, 5546–5557.

(36) Werner, H. J.; Knowles, P. J. A Second Order Multiconfiguration SCF Procedure with Optimum Convergence. *J. Chem. Phys.* **1985**, *82*, 5053–5063.

(37) Fdez. Galván, I.; Vacher, M.; Alavi, A.; Angeli, C.; Aquilante, F.; Autschbach, J.; Bao, J. J.; Bokarev, S. I.; Bogdanov, N. A.; Carlson, R. K.; Chibotaru, L. F.; Creutzberg, J.; Dattani, N.; Delcey, M. G.; Dong, S. S.; Dreuw, A.; Freitag, L.; Frutos, L. M.; Gagliardi, L.; Gendron, F.; Giussani, A.; González, L.; Grell, G.; Guo, M.; Hoyer, C. E.; Johansson, M.; Keller, S.; Knecht, S.; Kovačević, G.; Källman, E.; Li Manni, G.; Lundberg, M.; Ma, Y.; Mai, S.; Malhado, J. P.; Malmqvist, P. Å.; Marquetand, P.; Mewes, S. A.; Norell, J.; Olivucci, M.; Oppel, M.; Phung, Q. M.; Pierloot, K.; Plasser, F.; Reiher, M.; Sand, A. M.; Schapiro, I.; Sharma, P.; Stein, C. J.; Sørensen, L. K.; Truhlar, D. G.; Ugandi, M.; Ungur, L.; Valentini, A.; Vancoillie, S.; Veryazov, V.; Weser, O.; Wesołowski, T. A.; Widmark, P. O.; Wouters, S.; Zech, A.; Zobel, J. P.; Lindh, R. OpenMolcas: From Source Code to Insight. *J. Chem. Theory Comput.* **2019**, *15*, 5925–5964.

(38) Aquilante, F.; Autschbach, J.; Baiardi, A.; Battaglia, S.; Borin, V. A.; Chibotaru, L. F.; Conti, I.; De Vico, L.; Delcey, M.; Galván, I. F.; Ferré, N.; Freitag, L.; Garavelli, M.; Gong, X.; Knecht, S.; Larsson, E. D.; Lindh, R.; Lundberg, M.; Malmqvist, P. Å.; Nenov, A.; Norell, J.; Odelius, M.; Olivucci, M.; Pedersen, T. B.; Pedraza-González, L.; Phung, Q. M.; Pierloot, K.; Reiher, M.; Schapiro, I.; Segarra-Martí, J.; Segatta, F.; Seijo, L.; Sen, S.; Sergentu, D. C.; Stein, C. J.; Ungur, L.; Vacher, M.; Valentini, A.; Veryazov, V. Modern Quantum Chemistry with [Open]Molcas. *J. Chem. Phys.* **2020**, *152*, 214117.

(39) Barbatti, M.; Ruckenbauer, M.; Plasser, F.; Pittner, J.; Granucci, G.; Persico, M.; Lischka, H. Newton-X: A Surface-Hopping Program for Nonadiabatic Molecular Dynamics. *Wiley Interdiscip. Rev.: Comput. Mol. Sci.* **2014**, *4*, 26–33.

(40) Abiola, T. T.; Rodrigues, N. D. N.; Ho, C.; Coxon, D. J. L.; Horbury, M. D.; Toldo, J. M.; Do Casal, M. T.; Rioux, B.; Peyrot, C.; Mention, M. M.; Balaguer, P.; Barbatti, M.; Allais, F.; Stavros, V. G. New Generation UV-A Filters: Understanding Their Photodynamics on a Human Skin Mimic. *J. Phys. Chem. Lett.* **2021**, 12, 337–344.

(41) Shick, J. M.; Dunlap, W. C. Mycosporine-like Amino Acids and Related Gadusols: Biosynthesis, Accumulation, and UV-Protective Functions in Aquatic Organisms. *Annu. Rev. Physiol.* **2002**, *64*, 223–262.

(42) Suh, S. S.; Hwang, J.; Park, M.; Seo, H. H.; Kim, H. S.; Lee, J. H.; Moh, S. H.; Lee, T. K. Anti-Inflammation Activities of Mycosporine-like Amino Acids (MAAs) in Response to UV Radiation Suggest Potential Anti-Skin Aging Activity. *Mar. Drugs* **2014**, *12*, 5174–5187.

(43) Fazzi, D.; Barbatti, M.; Thiel, W. Unveiling the Role of Hot Charge-Transfer States in Molecular Aggregates via Nonadiabatic Dynamics. *J. Am. Chem. Soc.* **2016**, *138*, 4502–4511.

(44) Plasser, F.; Barbatti, M.; Aquino, A. J. A.; Lischka, H. Excited-State Diproton Transfer in [2,2′-Bipyridyl]-3,3′-Diol: The Mechanism Is Sequential, Not Concerted. *J. Phys. Chem. A* **2009**, *113*, 8490–8499.

(45) Grimme, S. Exploration of Chemical Compound, Conformer, and Reaction Space with Meta-Dynamics Simulations Based on Tight-Binding Quantum Chemical Calculations. *J. Chem. Theory Comput.* **2019**, *15*, 2847–2862.

(46) Bannwarth, C.; Ehlert, S.; Grimme, S. GFN2-XTB - An Accurate and Broadly Parametrized Self-Consistent Tight-Binding Quantum Chemical Method with Multipole Electrostatics and Density-Dependent Dispersion Contributions. *J. Chem. Theory Comput.* **2019**, *15*, 1652–1671.

(47) Crespo-Otero, R.; Barbatti, M. Spectrum Simulation and Decomposition with Nuclear Ensemble: Formal Derivation and Application to Benzene, Furan and 2-Phenylfuran. In *Highlights in Theoretical Chemistry*; Ornellas, F., João Ramos, M., Eds.; Springer, Berlin, Heidelberg: Berlin, **2014**; Vol. 4, pp 89–102.

(48) Swope, W. C.; Andersen, H. C.; Berens, P. H.; Wilson, K. R. A Computer Simulation Method for the Calculation of Equilibrium Constants for the Formation of Physical Clusters of Molecules: Application to Small Water Clusters. *J. Chem. Phys.* **1982**, *76*, 637–649.

(49) Tully, J. C. Molecular Dynamics with Electronic Transitions. *J. Chem. Phys.* **1990**, *93*, 1061–1071.

(50) Granucci, G.; Persico, M. Critical Appraisal of the Fewest Switches Algorithm for Surface Hopping. *J. Chem. Phys.* **2007**, *126*.

(51) Toldo, J. M.; do Casal, M. T.; Barbatti, M. Mechanistic Aspects of the Photophysics of UVA Filters Based on Meldrum Derivatives. *J. Phys. Chem. A* **2021**, *125*, 5499–5508.

(52) Cremer, D.; Pople, J. A. A General Definition of Ring Puckering Coordinates. *J. Am. Chem. Soc.* **1975**, *97*, 1354–1358.

(53) Spek, A. L. Single-Crystal Structure Validation with the Program PLATON. *J. Appl. Crystallogr.* **2003**, *36*, 7–13.
